# A link between magnesium-chelatase H subunit and sucrose nonfermenting 1 (SNF1)-related protein kinase SnRK2.6/OST1 in *Arabidopsis* guard cell signalling in response to abscisic acid

**DOI:** 10.1093/jxb/erv341

**Published:** 2015-07-13

**Authors:** Shan Liang, Kai Lu, Zhen Wu, Shang-Chuan Jiang, Yong-Tao Yu, Chao Bi, Qi Xin, Xiao-Fang Wang, Da-Peng Zhang

**Affiliations:** Center for Plant Biology, School of Life Sciences, Tsinghua University, Beijing 100084, China

**Keywords:** Abscisic acid signalling, *Arabidopsis thaliana*, guard cell, Mg-chelatase H subunit, SNF1-related protein kinase SnRK2.6/OST1, stomatal movement.

## Abstract

Magnesium-chelatase H subunit [CHLH/putative abscisic acid (ABA) receptor ABAR] positively regulates guard cell signalling in response to ABA, but the molecular mechanism remains largely unknown. A member of the sucrose nonfermenting 1 (SNF1)-related protein kinase 2 family, SnRK2.6/open stomata 1 (OST1)/SRK2E, which plays a critical role in ABA signalling in *Arabidopsis* guard cells, interacts with ABAR/CHLH. Neither mutation nor over-expression of the *ABAR* gene affects significantly ABA-insensitive phenotypes of stomatal movement in the *OST1* knockout mutant allele *srk2e*. However, *OST1* over-expression suppresses ABA-insensitive phenotypes of the *ABAR* mutant allele *cch* in stomatal movement. These genetic data support that OST1 functions downstream of ABAR in ABA signalling in guard cells. Consistent with this, ABAR protein is phosphorylated, but independently of the OST1 protein kinase. Two *ABAR* mutant alleles, *cch* and *rtl1*, show ABA insensitivity in ABA-induced reactive oxygen species and nitric oxide production, as well as in ABA-activated phosphorylation of a K^+^ inward channel KAT1 in guard cells, which is consistent with that observed in the *pyr1 pyl1 pyl2 pyl4* quadruple mutant of the well-characterized ABA receptor PYR/PYL/RCAR family acting upstream of OST1. These findings suggest that ABAR shares, at least in part, downstream signalling components with PYR/PYL/RCAR receptors for ABA in guard cells; though *cch* and *rtl1* show strong ABA-insensitive phenotypes in both ABA-induced stomatal closure and inhibition of stomatal opening, while the *pyr1 pyl1 pyl2 pyl4* quadruple mutant shows strong ABA insensitivity only in ABA-induced stomatal closure. These data establish a link between ABAR/CHLH and SnRK2.6/OST1 in guard cell signalling in response to ABA.

## Highlight

A sucrose nonfermenting 1 (SNF1)-related protein kinase 2, SnRK2.6/ open stomata 1 (OST1), which plays critical role in abscisic acid (ABA) signalling in *Arabidopsis* guard cells, interacts directly with, and functions downstream of, the magnesium-chelatase H subunit in guard cell signalling in response to ABA.

## Introduction

In higher plants, stomatal pores formed by a pair of guard cells play key roles in allowing photosynthesis and transpiration. Through controlling stomatal opening and closure, the plants regulate gas exchange and water loss, which is directly related to the turgor of guard cells. The change of turgor is modulated by the dynamic changes in intracellular concentration of ions and sugars (Archana *et al.*, 2011). Different channels and transporters are involved in ion flux across membranes mediated by phytohormone abscisic acid (ABA) signalling. In response to water deficit, ABA is synthesized and released from storage, and then serves as an endogenous messenger to promote stomatal closure.

In recent years, significant progress has been made in understanding ABA signalling of guard cells. Many signalling components have been identified, including a central regulator open stomata 1 (OST1, also known as SnRK2.6 or SRK2E), a member of the sucrose nonfermenting 1 (SNF1)-related protein kinase 2s family (Mustilli *et al.*, 2002; Yoshida *et al.*, 2002). Different from its homologues SnRK2.2 and SnRK2.3, which regulate mainly seed germination and seedling growth by activating ABA-responsive bZIP transcription factor ABF (Boudsocq *et al.*, 2004; Kobayashi *et al.*, 2004; Furihata *et al.*, 2006; Yoshida *et al.*, 2006; Fujii *et al.*, 2007; Fujii and Zhu, 2009; Fujii *et al.*, 2009), OST1 is preferentially expressed in guard cells, and the *OST1* gene mutant shows impaired ABA-induced stomatal closure, revealing that OST1 acts as a positive regulator of guard cell signalling in response to ABA (Mustilli *et al.*, 2002; Yoshida *et al.*, 2002). OST1 phosphorylates the inward K^+^ channel KAT1, and the C-terminal region of KAT 1is the direct phosphorylation target domain of OST1 (Sato *et al.*, 2009; Acharya *et al.*, 2013). Phosphorylation of KAT1 leads to inhibition of its activity to drive inward K^+^ flux, which is required for ABA-induced stomatal closure and inhibition of stomatal opening (Kwak *et al.*, 2001; Pandey *et al.*, 2007). ABA inhibition of inward K^+^ channels and light-induced stomatal opening are reduced in *ost1* mutants, while transgenic plants overexpressing *OST1* show ABA hypersensitivity in these responses, suggesting that OST1 negatively regulates KAT1 to induce stomatal closure and inhibit stomatal opening in response to ABA (Acharya *et al.*, 2013). These observations reveal that KAT1 is a node of the OST1-mediated ABA signalling cascades in guard cells. Slow (S-type) anion channel associated 1 (SLAC1) is another substrate of OST1, and the SLAC1 anion channel is activated by OST1 in a heterologous system (*Xenopus* oocytes) (Geiger *et al.*, 2009, 2010; Lee *et al.*, 2009, 2013; Brandt *et al.*, 2012; Acharya *et al.*, 2013). Genetic evidence supports that SLAC1, together with KAT1, plays critical roles in OST1-mediated guard cell signalling in response to ABA (Geiger *et al.*, 2009; Acharya *et al.*, 2013). In addition, OST1 phosphorylates a K^+^ uptake transporter KUP6 (Osakabe *et al.*, 2013), and regulates ABA activation of rapidly activating (QUAC1) anion currents in guard cells (Imes *et al.*, 2013), which may also be involved in the mechanism of OST1-mediated ABA signalling in guard cells.

ABA accumulation in guard cells triggers the generation of reactive oxygen species (ROS) (Pei *et al.*, 2000; Zhang *et al.*, 2001). ROS production is located downstream of OST1 in the ABA signalling of guard cells (Mustilli *et al.*, 2002; Acharya *et al.*, 2013), where ABA-activated OST1 interacts with and phosphorylates two NADPH oxidases, AtrbohD and AtrbohF, which play key roles in ABA-induced ROS generation in *Arabidopsis* guard cells (Kwak *et al.*, 2003; Acharya *et al.*, 2013). ROS serves as a second-messenger molecule regulating stomatal channels and transporters to mediate ABA signalling in guard cells. Exogenous ROS suppresses the inward K^+^ channel in *Vicia* guard cells (Zhang *et al.*, 2001). ROS also stimulates Ca^2+^ release from internal stores and influx across the plasma membrane, and then promotes stomatal closure (Pei *et al.*, 2000). Another second-messenger molecule—nitric oxide (NO)—also plays a positive role in ABA-induced stomatal closure (Neill *et al.*, 2002). The level of NO in guard cells increases dependently on the quick burst of ROS (Bright *et al.*, 2006), and NO may possibly function by targeting inward K^+^ and anion channels in the same way as ROS (Garcia-Mata *et al.*, 2003). NO also modulates guard cell signalling through the generation of nitrated cGMP (Joudoi *et al.*, 2013). A recent study reported that ABA-induced NO causes *S*-nitrosylation of OST1 and blocks its kinase activity, thereby regulating the ABA signalling pathway via negative feedback (Wang *et al.*, 2015).

Recent progress has established an ABA signalling pathway in guard cells from primary events to activation of different channels. Clade A protein phosphatase 2Cs (PP2Cs) bind to, dephosphorylate, and inhibit kinase activity of OST1, negatively regulating ABA signalling (Mustilli *et al.*, 2002; Yoshida *et al.*, 2006; Fujii *et al.*, 2009; Umezawa *et al.*, 2009; Vlad *et al.*, 2009, 2010; Cutler *et al.*, 2010). The START-domain family proteins PYR/PYL/RCARs—the best characterized cytosolic ABA receptors (Ma *et al.*, 2009; Park *et al.*, 2009; Santiago *et al.*, 2009; Cutler *et al.*, 2010; Nishimura *et al.*, 2010)—perceive the ABA signal, and bind to and inactivate PP2Cs, leading to release of OST1 from inhibition by PP2Cs (Mustilli *et al.*, 2002; Yoshida *et al.*, 2006; Fujii *et al.*, 2009; Umezawa *et al.*, 2009; Vlad *et al.*, 2009, 2010; Cutler *et al.*, 2010), activating downstream signalling components to finally relay the ABA signal to different ion channels, which modulates ion flux across guard cell membranes to induce stomatal closure (Kwak *et al.*, 2001; Pandey *et al.*, 2007; Geiger *et al.*, 2009, 2010; Lee *et al.*, 2009, 2013; Brandt *et al.*, 2012; Acharya *et al.*, 2013; Imes *et al.*, 2013; Osakabe *et al.*, 2013).

The magnesium-protoporphyrin IX chelatase large subunit (Mg-chelatase H subunit CHLH/putative ABA receptor ABAR) was reported to function as a candidate ABA receptor in the *Arabidopsis* chloroplast space, and an ABAR-mediated signalling pathway involving the chloroplast protein cochaperonin CPN20 and cytosolic-nuclear WRKY18/40/60 transcription repressors has been described (Shen *et al.*, 2006; Wu *et al.*, 2009; Shang *et al.*, 2010; Du *et al.*, 2012; Liu *et al.*, 2012; Liu *et al.*, 2013; Yan *et al.*, 2013; Zhang *et al.*, 2013, 2014; Wang and Zhang, 2014). Although whether it binds ABA remains controversial (Shen *et al.*, 2006; Müller and Hansson, 2009; Wu *et al.*, 2009; Tsuzuki *et al.*, 2011; Wang *et al.*, 2011; Du *et al.*, 2012), increasing evidence supports that CHLH/ABAR plays a crucial role in ABA signalling for major ABA responses including ABA-inhibited seed germination and post-germination growth, as well as ABA-induced stomatal closure and inhibition of light-induced stomatal opening. Four *abar* mutant alleles in *Arabidopsis*, *abar-2*, *abar-3*, *cch*, and *rtl1*, display impaired ABA responses (Shen *et al.*, 2006; Wu *et al.*, 2009; Tsuzuki *et al.*, 2011, 2013; Du *et al.*, 2012). A recent genetic screen has identified a pentatricopeptide repeat protein SOAR1 functioning as a hub of ABA signalling downstream of ABAR, and it was reported that *SOAR1* over-expression almost completely impairs ABA responses, suggesting that ABAR mediates a central ABA signalling pathway (Jiang *et al.*, 2014; Mei *et al.*, 2014; Wang and Zhang, 2014).

In different systems including *Arabidopsis*, *Nicotiana benthamiana*, and *Prunus persica* leaves, ABAR was shown to positively mediate guard cell signalling in response to ABA (Shen *et al.*, 2006; Legnaioli *et al.*, 2009; Wu *et al.*, 2009; Jia *et al.*, 2011; Tsuzuki *et al.*, 2011, 2013; Du *et al.*, 2012; Zhang *et al.*, 2013). Tsuzuki and co-workers (2013) recently showed that CHLH/ABAR mediates ABA inhibition of blue light (BL)-induced phosphorylation of H^+^-ATPase in *Arabidopsis* guard cells, suggesting that ABAR regulates not only ABA-induced stomatal closure but also ABA inhibition of BL-mediated stomatal opening. However, the molecular mechanism by which ABAR regulates stomatal movement in response to ABA remains largely unknown. In this study, SnRK2.6/OST1 is shown to be an interaction partner of ABAR, and functions downstream of ABAR in ABA signalling in *Arabidopsis* guard cells. Consistent with this point of view, ABAR was shown to share downstream signalling steps with the PYR/PYL/RCAR receptor for ABA in guard cells. These data establish a functional link between ABAR/CHLH and OST1 in guard cell signalling in response to ABA.

## Materials and methods

### Plant materials and growth conditions

*Arabidopsis thaliana* ecotype Columbia-0 (Col-0) was used to generate transgenic plants and as the wild-type control. To generate the SnRK2.6/OST1 (At4g33950) over-expression lines, the full-length sequence of OST1, amplified by PCR with the primers listed in Supplementary Table S1 (available at *JXB* online), was cloned into the binary vector pCAMBIA-1300-221, which, fused with the Myc-tags, was driven by the cauliflower mosaic virus (CaMV) *35S* promoter. The construct was introduced into *Agrobacterium tumefaciens*, and transformed to Col-0 plants to generate the OST1-over-expression lines (OST1OE). The OST1 levels were analysed by quantitative real-time PCR. ABAR-over-expression lines were generated by introducing an ABAR gene (At5g13630) fragment [encoding a truncated ABAR with amino acids (aa) 631–1381, named ABAR^631–1381^) into *Arabidopsis* ecotype Col-0 plants, where ABAR^631–1381^ was fused with GFP protein, and the construct was driven by *35S* promoter (Wu *et al.*, 2009). It was previously shown that this C-terminal half of ABAR tagged with GFP functions similarly to full-length ABAR in transgenic plants, leading to ABA hypersensitivity in the major ABA responses; the intensities of ABA-hypersensitive phenotypes of the C-terminal half of ABAR-expressing lines are similar to those of full-length ABAR-transgenic plants (Wu *et al.*, 2009). Therefore, the transgenic lines expressing this C-terminal half of ABAR were used to overexpress ABAR in this experiment. The cDNA isolation and transgenic manipulation were performed as previously described (Wu *et al.*, 2009).

The *cch* mutant and the *rtl1* mutant, two mutant alleles of the ABAR gene, were gifts from Dr J. Chory (The Salk Institute, La Jolla, CA, USA) and Dr T. Kinoshita (Nagoya University, Japan), respectively. The *pyr1 pyl1 pyl2 pyl4* quadruple ABA receptor knockout mutant (Park *et al.*, 2009) was a gift from Dr Cutler (University of California at Riverside, Riverside, CA, USA). The OST1 T-DNA insertion knockout mutant (SALK_008068) was obtained from *Arabidopsis* Biological Resource Center (ABRC), which was identified and described previously with the name *srk2e* (Yoshida *et al.*, 2002; Umezawa *et al.*, 2009). The *cch srk2e* double mutant, ABAR^631–1381^-over-expression lines under *srk2e* background (ABAR^631–1381^OE/*srk2e*), and OST1-over-expression lines under *cch* background (OST1OE-1/*cch*) were constructed by crossing. All the mutant lines were identified by PCR genotyping using the primers presented in Supplementary Table S1.

*Arabidopsis* seeds were disinfected and plated on MS medium (Sigma-Aldrich, St Louis, MO, USA) supplemented with 3% sucrose and 0.8% agar (pH 5.9), chilled for 3 d at 4 °C and transferred into a growth chamber at light intensity 80 μmol photons m^−2^ s^−1^ or into compost at 120 μmol photons m^−2^ s^−1^ using cool white fluorescent lamps under a 16h light/8h dark photoperiod and 60% relative humidity.

### Yeast two-hybrid assays

Interaction between ABAR and OST1 was assayed by a yeast two-hybrid system as described by the manufacturer (Clontech, Mountain View, CA, USA). Three truncated ABAR proteins—the C-terminal half of ABAR (aa 692–1381, ABAR_c690_), the N-terminal half of ABAR (aa 1–691, ABAR_n691_), and the middle section of ABAR (aa 692–941, ABAR_c250_)—were fused to GAL4 binding domain (BD) in the pGBKT7 vector. The full-length sequence of OST1 was cloned into the prey plasmid pGADT7 with the DNA activation domain (AD). The primers used for constructing the related plasmids are listed in Supplementary Table S1. The different combinations of constructs were co-transformed to yeast strain AH109 and tested on SD screening medium as indicated for 5–7 d at 30 °C. To test α-galactosidase (α-Gal) activity of transformed yeast cells, substrate ρ-nitrophenyl-α-d-galactoside was dropped onto the transformed yeast cells to detect the colour changes according to the manufacturer’s protocols. The yeast co-transformed with the construct pairs AD plus BD-ABAR_c690_ and BD plus AD-OST1, were taken as negative controls. The yeast co-transformed with the construct pair BD-53 plus AD-T was taken as a positive control. For the CoIP assays in yeast, yeast strains were cultured on SD4- medium to OD_600_ 1.0 at 30 °C, and then cells were harvested and lysed with an extraction buffer containing 50mM HEPES, pH 7.4, 10mM EDTA, 0.1% (v/v) TritonX-100, 1mM phenylmethylsulphonyl fluoride (PMSF), and 5 μg/ml protein inhibitor cocktail (Roche, Mannheim, Germany). Total protein was pre-cleared with the protein A/G plus beads (Santa Cruz Biotechnology, Dallas, TX, USA) and was divided into two parts. One was incubated with mouse anti-HA-tag antibody (MBL, Nagoya, Japan) and the other was incubated with pre-immune serum (MBL, Nagoya, Japan) for 1h. After incubation, the protein A/G plus beads were added into the buffer and incubation continued at 4 °C for another 4h. The beads were washed twice with buffer A containing 50mM Tris (pH 8.0), 150mM NaCl, and 0.1% (v/v) Triton X-100 and twice with buffer B containing 50mM Tris (pH 8.0) and 0.1 % (v/v) Triton X-100, and then resuspended in protein loading buffer. The immunoprecipitates were separated on a 10 % SDS-PAGE and analysed by immunoblotting with anti-Myc serum.

### Luciferase complementation imaging assay

The luciferase complementation imaging (LCI) assay was used to further confirm ABAR and OST1 interaction in *N. benthamiana* leaves according to previously described procedures (Chen *et al.*, 2008). The firefly luciferase (Luc) enzyme was divided into the N-terminal part (NLuc) and C-terminal part (CLuc). ABAR and OST1 were cloned into pCAMBIA-NLuc vector fused with NLuc and pCAMBIA-CLuc vector fused with CLuc, respectively. The primers used for constructing the related plasmids are listed in Supplementary Table S1. The constructs were transformed into *A. tumefaciens* strain GV3101. Using the *A. tumefaciens*-mediated transformation with equal concentrations and volumes, different combinations of constructs were introduced to the fully expanded leaves of the 7-week-old *N. benthamiana* plants by a needleless syringe. The amounts of the constructs were kept the same among treatments and controls for each group of assays. After infiltration, plants were placed with 16h light/8h dark for 48h at 24 °C. The Luc activity was observed by a cooled CCD imaging apparatus (Andor iXon, Andor Technology, Belfast, UK).

### Preparation of recombinant proteins in *Escherichia coli*

To prepare recombinant OST1 and truncated KAT1 protein, the full-length ORF of OST1 and a KAT1 fragment encoding the truncated KAT1 (corresponding to the C-terminal region covering aa 301–677) were isolated using the primers listed in Supplementary Table S1, and cloned into pET-48b (+) vector (Novagen, Madison, WI, USA). The recombinant plasmids were expressed in *E. coli* strain BL21(DE3) as His-tagged fusion proteins. The *E. coli* strains were grown at 37 °C in LB medium until the OD_600_ of the cultures was 0.8. Protein expression was induced by the addition of IPTG to a final concentration of 0.5mM at 16 °C. After 16h incubation, the cells were harvested by centrifugation at 10 000 *g* and resuspended in the extracting buffer (25ml) containing 10mM Tris-HCl (pH 7.5), 200mM NaCl, and 10% glycerol, and 5 μg ml^−1^ protein inhibitor cocktail (Roche). The mixture was subjected to sonication three times until the cells were lysed. The lysate was centrifuged at about 10 000 *g*, and the supernatant was transferred to a purification column. Proteins were purified according to manufacturer’s instructions (Novagen, Madison, WI, USA) using Ni-NTA agarose, and the eluted protein was dialyzed against the extracting buffer. To prepare the recombinant truncated ABAR protein, the sequence fragment encoding a truncated ABAR harbouring aa 681–1381 was amplified by PCR with the primers listed in Supplementary Table S1, and cloned into pGEX-4T-1 (GE Healthcare, Piscataway, NJ, USA) with GST-tag. The truncated ABAR protein was expressed by inducing with IPTG in *E. coli* strain BL21(DE3) with the same procedures as described above, and purified according to manufacturer’s instructions (GE Healthcare, Piscataway, NJ, USA) using Sepharose 4B. Protein concentration was determined by the method of Bradford (1976) with BSA as a standard.

### GST-pull-down assay

GST-pull-down assays were conducted to test further the interaction of the C-terminal half of ABAR protein with OST1. The recombinant OST1 protein fused with His tag and the C-terminal half of ABAR protein (aa 681–1381) fused with GST-tag were prepared as described above in *E. coli*. The C-terminal half of ABAR protein fused with GST-tag (1 μg) or GST protein alone was added into *E. coli* cell lysate expressing His-tagged OST1 protein. Samples were incubated rotating at 4 °C for 12h with glutathione-sepharose 4B beads, which bind GST. GST pellets, collected by centrifugation at 3000 *g*, were washed five times with 1ml of the extracting buffer containing 10mM Tris-HCl (pH 7.5), 200mM NaCl, and 10% glycerol, and 5 μg ml^−1^ protein inhibitor cocktail (Roche). After the wash, GST-bound proteins were resuspended in protein loading buffer. Samples were separated on a 12 % SDS-PAGE and analysed by immunoblotting with anti-His serum.

### CoIP in plants

The CoIP assay was performed essentially as described previously (Shang *et al.*, 2010). Myc-tagged OST1 over-expression lines were used to perform the CoIP assay. The plant total protein was prepared using extraction buffer (3mg/ml) containing 50mM Tris-HCl (pH 7.4), 10 % glycerol (v/v), 1mM EDTA, 150mM NaCl, 0.1 % Triton X-100 (v/v), 1mM PMSF, and 5 μg/ml protein inhibitor cocktail (Roche). Total protein was pre-cleared with the protein A/G plus beads (Santa Cruz Biotechnology, Dallas, TX, USA) and divided into two parts; one incubated with mouse anti-Myc-tag antibody (MBL, Nagoya, Japan) and the other incubated with pre-immune serum (MBL, Nagoya, Japan) for 1h. After incubation, the protein A/G plus beads were added into the buffer and incubation continued at 4 °C for another 4h. The beads were washed five times extensively with extraction buffer and then resuspended in protein loading buffer. The immuno-precipitates were separated on a 10 % SDS-PAGE and analysed by immunoblotting with anti-ABAR serum. The anti-ABAR serum was produced as described previously (Shen *et al.*, 2006; Wu *et al.*, 2009; Shang *et al.*, 2010).

### Stomatal movement assay

The stomatal movement assay was performed essentially as described previously (Shen *et al.*, 2006; Wu *et al.*, 2009; Shang *et al.*, 2010). Mature rosette leaves of 4-week-old plants were used for the stomatal aperture assay, which was conducted under normal air. To assay ABA-induced stomatal closure, leaves were immersed in a solution containing 50mM KCl and 10mM MES-KOH (pH 6.5), and exposed to a halogen cold light source for 3h. Subsequently, (±)ABA or an equal amount of ethanol for dissolving ABA (as the ABA-free controls) at different concentrations was added into the buffer. Stomatal apertures were measured 2.5h after ABA treatment. To assay ABA-inhibited stomatal opening, leaves were immersed in the same solution as described above in the dark for 12h before they were transferred to the cold light for 2.5h in the presence of ABA, and then apertures were determined. Five plants for each genotype (Col, *pyr1 pyl1 pyl2 pyl4* quadruple mutant, and *cch* and *rtl1* mutants) and one mature rosette leaf from each plant was sampled for the stomatal aperture assay, and five leaves were used in total for each experiment. More than 20 stomata were measured for each leaf, and so more than 80 stomata were measured for each experiment. The experiment was conducted line- and treatment-blind, and repeated independently three times with similar results.

### Water loss and drought assays

For the water loss assay, rosette leaves were detached from the roots and placed on a plastic dish. Water loss was evaluated by weighing excised leaves at the indicated times under room temperature conditions. For drought treatment, plants were grown on soil for ~5 d and then drought was imposed by withdrawing irrigation until the lethal effect of dehydration was observed on the majority of the plants, whereas the other half were grown under a standard irrigation regime as a control.

### Measurement of ROS and NO production

The production of ROS and NO in guard cells was estimated using the fluorescence indicators 2′,7′-dichlorodihydrofluorescein diacetate (H_2_DCF-DA) and diaminofluorescein-FM diacetate (DAF-FM-DA) (Sigma-Aldrich, St Louis, MO, USA), respectively. The epidermal strips were pre-incubated for 2h under conditions promoting stomatal opening in the MES-Tris buffer (pH 6.15; pre-incubation buffer) supplemented with 0 (ethanol, as a control) or 10 μM (±)ABA, and were incubated in buffer containing 50mM Tris-HCl (pH 7.2) with 50 μM H_2_DCF-DA or 20mM HEPES-NaOH buffer (pH 7.4) with 10 μM DAF-FM-DA in the dark for 20min. After the treatment, the epidermal tissues were washed with the same pre-incubation buffer to remove excess dye. Examinations of peel fluorescence were performed using a fluorescence microscopy (Zeiss, Oberkochen, Germany; excitation, 488nm; emission, 525nm). All pictures were taken under the same exposure intensity to reduce the influence of the background intensities. Image J software was used to calculate the corrected average optical density (OD) to represent fluorescence intensities, which are the result of the guard cell OD minus background OD.

### Quantitative real-time PCR analysis

Total RNA was extracted from 2-week-old seedlings with the RNasy plant mini kit (Qiagen, Hilden, German) according to the manufacturer’s instructions. Single-strand cDNA was synthesized by using total RNA (2 µg) with the M-MLV reverse transcriptase (NEB, Ipswich, MA, USA). Quantitative real-time PCR (qRT-PCR) was performed using the CFX96^TM^ Real-Time System of C1000^TM^ Thermal Cycler (Bio-Rad, Hercules, CA, USA) and SYBR Premix Ex Taq (TaKaRa Bio, Dalian, China) with the program: 5min at 94 °C and then 30 cycles of 5sec at 94 °C, 30sec at 60 °C. ACTIN2/8 gene was used as an internal control. Primers for qRT-PCR are listed in Supplementary Table S1. The qRT-PCR was performed in triplicate and means of the three biological repeats were calculated to represent gene expression level.

### Phos-tag SDS-PAGE assay to test phosphorylation

SDS-PAGE was performed according to the method of Laemmli (1970). The Phos-tag ligand AAL-107 was purchased from Wako Pure Chemical Industries (Osaka, Japan). Mn^2+^-Phos-tag SDS-PAGE was performed according to manufacturer’s guidebook. The acrylamide pendant Phos-tag ligand with final concentration of 50 μM and two equivalents of MnCl_2_ were added into the gel before polymerization. Electrophoresis was performed at 30 mA until the bromophenol blue dye reached the bottom of the separating gel. Immunoblotting was performed according to previously described procedures (Shen *et al.*, 2006; Wu *et al.*, 2009) with anti-His-tag (MBL, Nagoya, Japan) or anti-CHLH/ABAR serum for detecting corresponding target proteins.

To assay the phosphorylation of ABAR, 3-week-old plants of Col and *srk2e* were treated with ABA-free (-ABA) or ABA-containing solution [50 μM (±)ABA] for 90min, then the total protein was prepared from these plants using extraction buffer containing 50mM Tris-HCl (pH 8.0), 5mM MgCl_2_, 0.1mM ZnCl_2_, 0.02% Triton X-100 (v/v), 100 μM PMSF, and 5 μg ml^−1^ protein inhibitor cocktail. The total protein was used for Mn^2+^-Phos-tag SDS-PAGE assay.

To assay the His-tagged phosphorylation of the C-terminal domain of the KAT1 protein, the recombinant truncated KAT1 protein containing the C-terminal region His301–Asn677 was treated with alkaline phosphatase (AP, Sigma-Aldrich, St Louis, MO, USA) in a 50 mM-Tris-HCl buffer (pH 8.5) containing 1mM MgCl_2_ for 6h at 37 °C, and purified using Ni-NTA beads. After purification, the eluted protein was dialyzed against AP reaction buffer. The total protein used for the KAT1 phosphorylation was prepared from 3-week-old plants of Col, quadruple, and *cch* mutants treated with the ABA-free (−ABA) or ABA-containing solution [50 μM (±)ABA] for 90min. The buffer used for extracting the total protein contained 50mM Tris-HCl (pH 8.0), 1mM MgCl_2_, 0.1mM ZnCl_2_, 1mM NaF, 0.02% TritonX-100 (v/v), and 5 μg ml^−1^ protein inhibitor cocktail. The total protein (30 μg) from the different genotypes was incubated in the medium containing the purified AP treatment KAT1^301–677^ protein (as a substrate, 2 μg) in the presence of 50 μM ATP for 3h at room temperature. The reaction mixture was analysed by Mn^2+^-Phos-tag SDS-PAGE assay.

## Results

### ABAR interacts with OST1

**Fig. 1.**
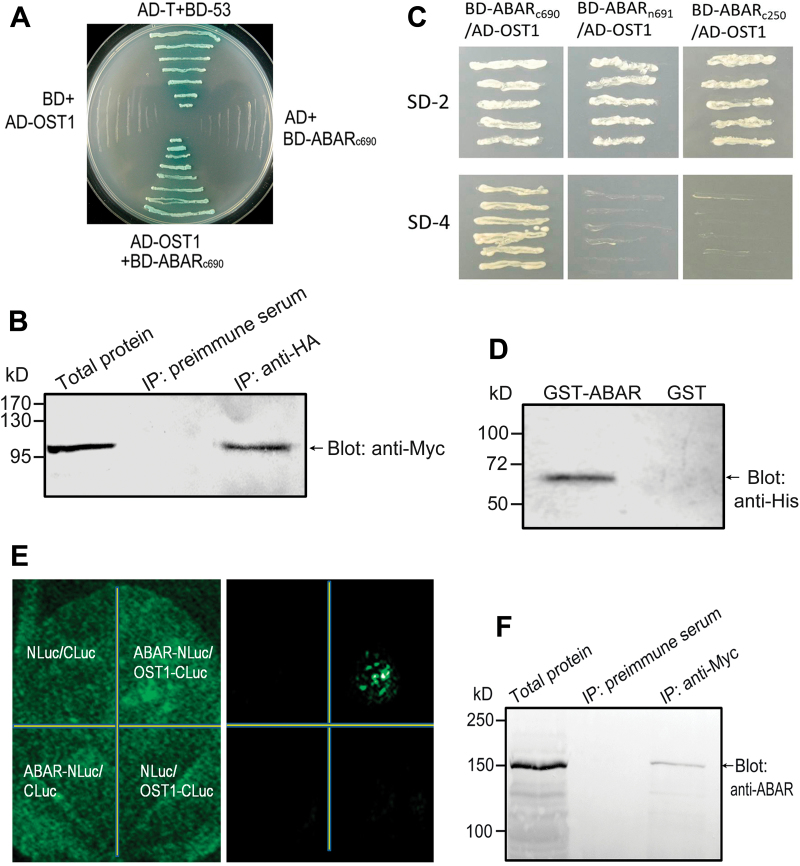
ABAR/CHLH physically interacts with OST1/SnRK2.6/SRK2E. (A) Assays of yeast growth in SD4-drop-out medium (lacking Leu, Trp, His, and Ade) to test for an interaction between ABAR and OST1. BD-ABAR_c690_, truncated ABAR protein [the C-terminal fragment: aa 692–1381 (690 aa)] fused with BD domain; AD-OST1, full-length OST1 fused with AD domain. The yeast co-transformed with the construct pairs AD plus BD-ABAR_c690_ and BD plus AD-OST1, were taken as negative controls. The yeast co-transformed with the construct pair BD-53 plus AD-T was taken as a positive control. Note that the yeast co-transformed with the construct pair AD-OST1 plus BD-ABAR_c690_ was able to grow in the SD4-drop-out medium. (B) Co-IP assays in yeast cells. Myc-ABAR and HA-OST1 were coimmunoprecipitated from yeast total proteins. Immunoprecipitation with pre-immune serum was taken as a negative control. (C) Test of the interaction of three different regions of ABAR with OST1 in the yeast two-hybrid system. ABAR_c690_; ABAR_n691_, N-terminal region of ABAR (aa 1–691); ABAR_c250_, the middle section of ABAR [aa 692–941, (250 aa)]. The yeast were co-transformed with the construct pairs BD-ABAR_c690_/AD-OST1, BD-ABAR_n691_/AD-OST1, and BD-ABAR_c250_/AD-OST1, and only the yeast co-transformed with the construct pair BD-ABAR_c690_/AD-OST1 was able to grow on the SD-4 medium (lacking Leu, Trp, His, and Ade). (D) GST-pull down assay to further test the interaction of the C-terminal half of ABAR with OST1. The GST-tagged C-terminal half of ABAR protein (GST-ABAR) pulled down the His-tagged OST1, which was detected by western blot analysis with anti-His, while GST alone did not pull down His-tagged OST1, which was taken as a negative control. (E) LCI to test the interaction of ABAR with OST1. The *N. benthamiana* leaves were co-transformed by infiltration using a needleless syringe with construct pairs as indicated in the left panel (Bright field). NLuc and CLuc, N-terminal and C-terminal half of the luciferase (Luc), respectively. ABAR-NLuc, full-length ABAR fused with NLuc; OST1-CLuc, full-length OST1 fused with CLuc. The right panel shows the luciferin fluorescence of the treated leaf. (F) ABAR co-immunoprecipitates with Myc-tagged OST1 protein from transgenic *Arabidopsis* (expressing Myc-tagged OST1) total proteins. Immunoprecipitation with pre-immune serum was taken as a negative control.

To identify the interaction partners of ABAR, a fragment encoding the C-terminus of ABAR (aa 692–1381, ABAR_c690_) was used as a bait to screen the *Arabidopsis* cDNA library in a yeast two-hybrid system, and OST1 was identified as a candidate. Further assays were performed to confirm the interaction between ABAR and OST1. Full-length OST1 was cloned to pGADT7 fused with the AD, and the ABAR fragment encoding ABAR_c690_ was cloned into pGBKT7 fused with the BD. The yeast cells co-transformed with the construct pair AD-OST1 plus BD-ABAR_c690_ or BD-53 plus AD-T (a positive control) were able to grow in the SD4-drop-out medium (lacking Leu, Trp, His, and Ade) and turned blue in the presence of α-Gal (Fig. 1A), while the yeast cells co-expressing the construct pairs AD plus BD-ABAR_c690_ and BD plus AD-OST1, taken as negative controls, were not able to grow in the SD4-drop-out medium (Fig. 1A), indicating that ABAR interacts with OST1 and that the interaction detected in this yeast system is specific and reliable. Co-IP assays in the yeast cells confirmed the interaction of ABAR with OST1 in the yeast system (Fig. 1B). The further experiments showed that, whereas ABAR_c690_—the C-terminal half of ABAR—is an interaction domain, neither the N-terminal region of ABAR (aa 1–691, ABAR_n691_) nor the middle section of ABAR (aa 692–941, ABAR_c250_) interacts with OST1 (Fig. 1C). The interaction of the C-terminal half of ABAR with OST1 was further confirmed in a pull down assay with the recombinant C-terminal half of ABAR and OST1 proteins (Fig. 1D), consistent with the idea that ABAR interacts with OST1 in the cytosolic space through its C-terminal half.

The LCI assay in *N. benthamiana*, where ABAR and OST1 were fused with the N-terminus of Luc (NLuc) and the C-terminus of Luc (CLuc), respectively, showed that the ABAR-NLuc and OST1-CLuc co-expressed leaves displayed significant Luc activity (fluorescence), but no Luc activity was detected in the leaves co-infiltrated with three negative-control pairs—NLuc and CLuc, ABAR-NLuc and CLuc, or NLuc and OST1-CLuc (Fig. 1E). In the CoIP assays in *Arabidopsis*, the anti-Myc-tag serum pulled down ABAR protein from the total proteins extracted from the Myc-tagged OST1-over-expression lines, indicating that ABAR co-immunoprecipitates with Myc-tagged OST1 protein. No immuno-signal was detected with anti-ABAR serum in the pre-immune serum-coimmunoprecipitated sample (Fig. 1F). These results indicate that ABAR interacts with OST1 *in vivo*.

The data from an assay with the surface plasmon resonance (SPR) technique showed that the C-terminal half of ABAR (aa 681–1381) binds OST1 with a saturation curve typical for protein–protein interaction and the signal strength was dose-dependent (Supplementary Fig. S1A, B available at JXB online), suggesting that ABAR may directly interact with OST1 independently of other proteins.

### Neither mutation nor over-expression of ABAR gene significantly changes ABA-insensitive phenotypes of the OST1 knockout mutant *srk2e*

**Fig. 2.**
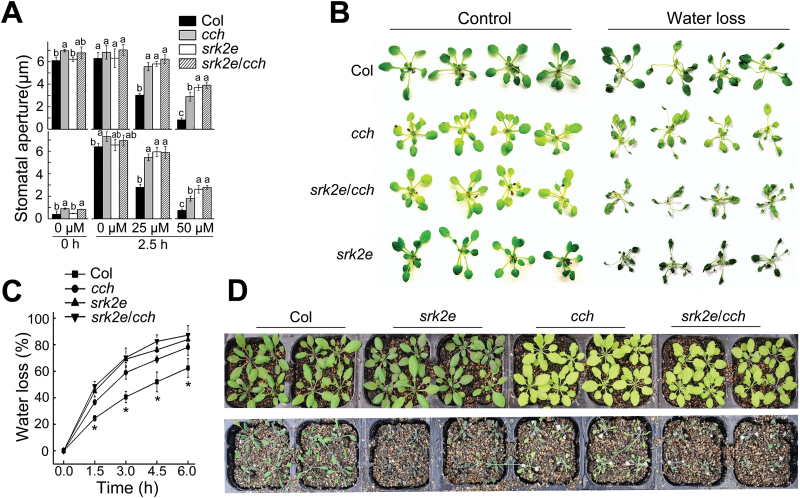
Genetic interaction between ABAR/CHLH and OST1/SnRK2.6/SRK2E: mutation of the *ABAR* gene does not significantly enhance ABA insensitivity of the *OST1*/*SnRK2.6*/*SRK2E* knockout mutant allele *srk2e* in stomatal movement. (A) ABA-induced stomatal closure (top) and inhibition of stomatal opening (bottom) in wild-type Col, *cch*, and *srk2e* single mutants and *srk2e cch* double mutant. *cch* is a mutant allele in the *ABAR* gene. Values are means ± SE from three independent experiments, and different letters indicate significant differences at *P*<0.05 (Duncan’s multiple range test) when comparing values within the same ABA concentration. *n*≥60 apertures per experiment. (B) Status of the detached leaves of the Col, *cch*, *srk2e*, and *srk2e cch*, which were subjected to a 6-h period water loss assay. (C) Water loss rates during a 6-h period from the detached leaves of the different genotypes described in (B). Values are means ±SE from three independent experiments. *P<0.05 (Duncan’s multiple range test) when comparing values within the same time point. (D) Water loss assays with young seedlings of the Col, *cch*, *srk2e*, and *srk2e cch*. Plants were well watered for 5 d then drought-stressed by withholding water for 15 d (bottom). Top panel shows the well watered control plants. The entire experiment was replicated three times with similar results.

Next, it was tested whether ABAR and OST1—two positive regulators in guard cell signalling in response to ABA (Mustilli *et al.*, 2002;Yoshida *et al.*, 2002; Shen *et al.*, 2006; Legnaioli *et al.*, 2009; Wu *et al.*, 2009; Jia *et al.*, 2011; Tsuzuki *et al.*, 2011, 2013; Du *et al.*, 2012; Zhang *et al.*, 2013)—functionally interact. It was observed that stomatal apertures of both the *cch* single and *srk2e cch* double mutants was slightly larger than that of wild-type Col plants under the exogenous ABA-free conditions, but the differences were not always statistically significant. This seems to be the result of the nuance of changes in environmental conditions among the independent repetitions, which might cause differences in guard cell responses. The intensity of the ABA-insensitive phenotypes of the *srk2e cch* double mutant in ABA-induced stomatal closure and ABA-inhibited stomatal opening was shown to be comparable with that of both *cch* and *srk2e* single mutants with 25 μM (±)ABA application, while in a higher ABA concentration [50 μM (±)ABA], this ABA-insensitive intensity of the *srk2e cch* double mutant was stronger than that of the *cch* single mutant and remained similar to that of the *srk2e* single mutant (Fig. 2A). The detached leaves of the three mutant plants lost water faster than those of wild-type Col plants, where the double mutant *srk2e cch* showed the highest loss rate, followed by *srk2e* and *cch* (Fig. 2B, C). The sensitivities to drought of these mutants showed similar trends to the water loss rates of their detached leaves (Fig. 2D). The observations of the dehydration assays with both the detached leaves and whole plants are consistent with those of stomatal movement.

**Fig. 3.**
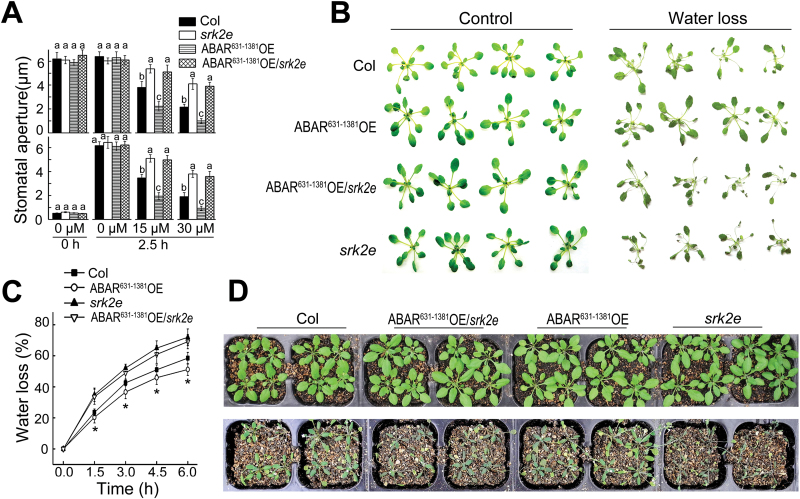
Genetic interaction between ABAR/CHLH and OST1/SnRK2.6/SRK2E: *ABAR* over-expression does not significantly affect ABA-insensitive phenotypes of the *srk2e* mutant in stomatal movement. (A) ABA-induced stomatal closure (top) and inhibition of stomatal opening (bottom) in wild-type Col, *srk2e* sigle mutant, ABAR^631–1381^ over-expression line under Col backgroud (ABAR^631–1381^OE), and ABAR^631–1381^ over-expression line under *srk2e* backgroud (ABAR^631–1381^OE/*srk2e*). ABAR^631–1381^ is a truncated form of ABAR (aa 631–1381). Values are means ±SE from three independent experiments, and different letters indicate significant differences at *P*<0.05 (Duncan’s multiple range test) when comparing values within the same ABA concentration. *n*≥60 apertures per experiment. (B) Status of the detached leaves of the Col, *srk2e*, ABAR^631–1381^OE, and ABAR^631–1381^OE/*srk2e*, which were subjected to a 6-h period water loss assay. (C) Water loss rates during a 6-h period from the detached leaves of the different genotypes described in (B). Values are means ±SE from three independent experiments. *P<0.05 (Duncan’s multiple range test) when comparing values within the same time point. (D) Water loss assays with young seedlings for the Col, *srk2e*, ABAR^631–-1381^OE, and ABAR^631–1381^OE/*srk2e*. Plants were well watered for 5 d then drought-stressed by withholding water for 17 d (bottom). Top panel shows the well watered control plants. The entire experiment was replicated three times with similar results.

It has been known that the over-expression of either the C-terminal half of ABAR (aa 631–1381) in whole Col plants (ABAR^631–1381^OE, Wu *et al.*, 2009) or the full-length ABAR specifically in guard cells (Tsuzuki *et al.*, 2013) confers ABA hypersensitivity in ABA-mediated stomatal response. ABAR^631–1381^- over-expression lines were created under the *srk2e* mutant background by crossing (ABAR^631–1381^OE/*srk2e*, Supplementary Fig. S2), which did not suppress the *srk2e* mutant phenotype, but showed an ABA-insensitive phenotype, like the *srk2e* background, in ABA-induced stomatal closure and ABA-inhibited stomatal opening (Fig. 3A). In addition, whereas over-expression of ABAR^631–1381^ in the Col background improved dehydration tolerance, over-expression of the same truncated ABAR under *srk2e* mutant did not affect the dehydration overly sensitive phenotypes of the *srk2e* mutant (Fig. 3B–D), which is consistent with the data from the investigation on stomatal movement in response to ABA (Fig. 3A).

### Over-expression of OST1suppresses ABA-insensitive phenotypes of the ABAR mutant *cch*

**Fig. 4.**
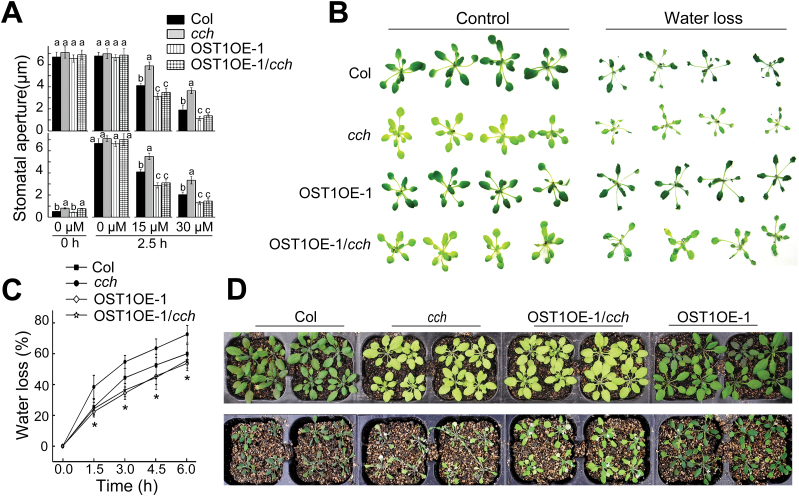
Genetic interaction between ABAR/CHLH and OST1/SnRK2.6/SRK2E: *OST1* over-expression suppresses ABA-insensitive phenotypes of the *cch* mutant in stomatal movement. (A) ABA-induced stomatal closure (top) and inhibition of stomatal opening (bottom) in wild-type Col, *cch* mutant, OST1 over-expression line under Col background (OST1OE-1), and OST1 over-expression line under *cch* background (OST1OE-1/*cch*). Values are means ±SE from three independent experiments, and different letters indicate significant differences at *P*<0.05 (Duncan’s multiple range test) when comparing values within the same ABA concentration. *n*≥60 apertures per experiment. (B). Status of the detached leaves of the Col, *cch*, OST1OE-1, and OST1OE-1/*cch*, which were subjected to a 6-h period water loss assay. (C) Water loss rates during a 6-h period from the detached leaves of the different genotypes described in (B). Values are means ±SE from three independent experiments. *P<0.05 (Duncan’s multiple range test) when comparing values within the same time point. (D) Water loss assays with young seedlings of the Col, *cch*, OST1OE-1, and OST1OE-1/*cch*. Plants were well watered for 5 d then drought-stressed by withholding water for 14 d (bottom). Top panel shows the well watered control plants. The entire experiment was replicated three times with similar results.

To further investigate functional interaction between ABAR and OST1, OST1-over-expression lines were generated in which the OST1 protein was Myc-tagged (Supplementary Fig. S3A). The OST1-transgenic lines displayed ABA-hypersensitive response in stomatal movement as previously reported (Acharya *et al.*, 2013), and the intensities of the ABA-hypersensitive phenotypes were positively correlated with the OST1-expression levels (Supplementary Fig. S3B). The OST1 over-expression line (OST1OE-1) was crossed with the *cch* mutant to create an OST1 over-expression line under the *cch* mutant background (OST1OE-1/*cch*). This OST1OE-1/*cch* line showed ABA-hypersensitive phenotypes in ABA-induced stomatal closure and ABA-inhibited stomatal opening like the OST1 over-expression line, which suppresses ABA-insensitive phenotypes of the *cch* mutant (Fig. 4A). The OST1OE-1 showed dehydration tolerance in contrast to *cch* that is dehydration hypersensitive, and the OST1OE-1/*cch* line showed dehydration tolerance like the OST1OE-1 line in the assays in both detached leaves and whole plants (Fig. 4B–D), which is consistent with the data from the assays of stomatal movement in response to ABA (Fig. 4A).

### Both *cch* and *rtl1* mutations in the *ABAR* gene impair ABA-induced ROS and NO production like the *pyr1 pyl1 pyl2 pyl4* quadruple mutant

**Fig. 5.**
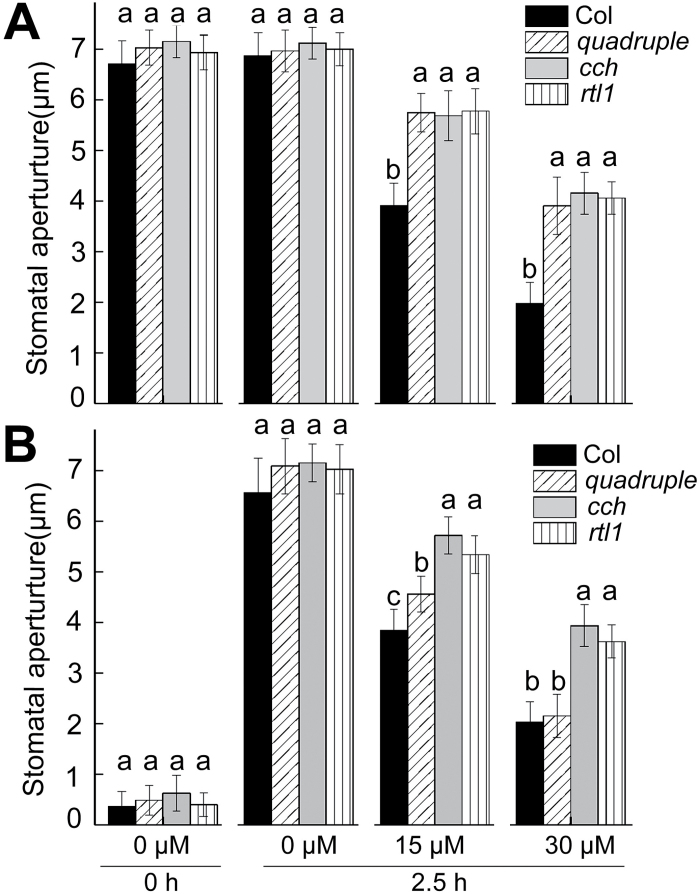
ABA-induced stomatal closure (A) and ABA-inhibited light-induced stomatal opening (B) in the wild-type Col, the *pyr1 pyl1 pyl2 pyl4* quadruple mutant (*quadruple*), and two mutant alleles of the *ABAR/CHLH* gene (*cch* and *rtl1*). Values are means ±SE from three independent experiments, and different letters indicate significant differences at *P*<0.05 (Duncan’s multiple range test) when comparing values within the same ABA concentration. *n*≥60 apertures per experiment.

To assess a possible mechanism by which ABAR and OST1 interact in ABA signalling, ABA-induced ROS and NO production in guard cells was measured in the ABAR gene mutants, using the *pyr1 pyl1 pyl2 pyl4* quadruple mutant of the PYR/PYL/RCAR gene family, encoding the cytosolic receptors for ABA upstream of OST1 as a control. It was first confirmed that the two mutant alleles, *cch* and *rtl1*, of the ABAR gene show both strong ABA-insensitive phenotypes in ABA-induced stomatal closure and inhibition of light-induced stomatal opening, while the *pyr1 pyl1 pyl2 pyl4* quadruple mutant shows strong ABA-insensitive phenotypes only in ABA-induced stomatal closure but not in ABA inhibition of light-induced stomatal opening (Fig. 5). The *pyr1 pyl1 pyl2 pyl4* quadruple mutant showed slight ABA-insensitive phenotypes in ABA inhibition of stomatal opening with 15 μM (±)ABA application, and the phenotypic intensity was significantly weaker than that of *cch* or *rtl1*, while in a higher ABA concentration [30 μM (±)ABA], the *pyr1 pyl1 pyl2 pyl4* quadruple mutant showed a wild-type ABA response in light-induced stomatal opening (Fig. 5), which is largely consistent with previous observations (Yin *et al*., 2013).

**Fig. 6.**
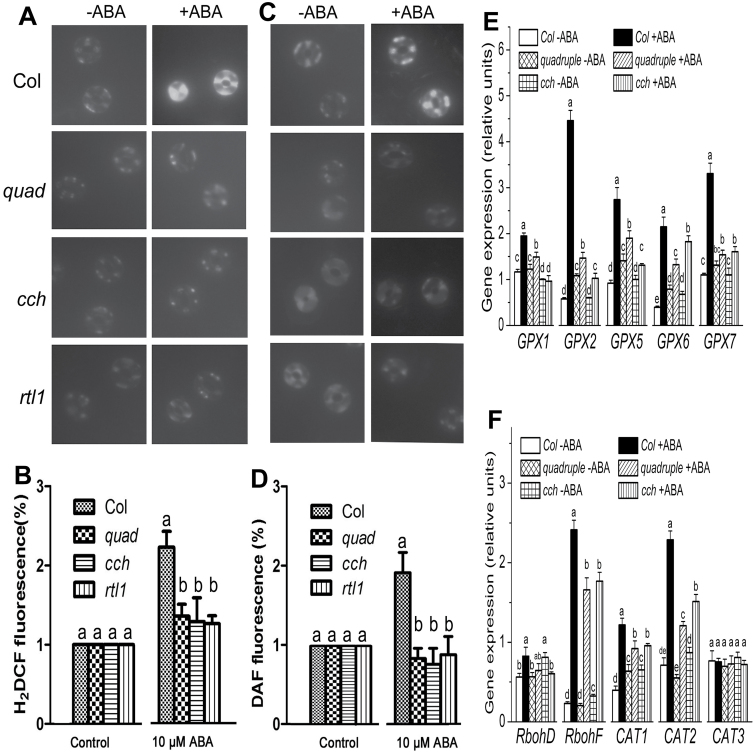
ABA-induced ROS and NO production and changes in the expression of some ROS-metabolism genes in guard cells of different genotypes. ROS production in response to ABA [10 μM (±)ABA, 20min treatment] was examined by H_2_DCF-DA imaging (A) and also the relative H_2_DCF fluorescence levels were recorded (B). NO production in response to ABA [10 μM (±)ABA, 20min treatment] was examined by diaminofluorescein (DAF) fluorescence imaging (C) and also the relative DAF fluorescence levels were recorded (D). The experiment was replicated three times with the similar results. The relative fluorescence levels are normalized relative to the control (−ABA) taken as 1. (E) and (F) show ABA-induced changes in the expression of some ROS-metabolism genes in guard cells of different genotypes. Two-week-old seedlings, sprayed with 50 μM (±)ABA or ABA-free solution (as a control), were sampled for RNA extraction 2.5h after the ABA application. The expression of the related genes was assayed by real-time PCR. Values in B, D, E, and F are means ±SE from three independent experiments, and different letters indicate significant differences at *P*<0.05 (Duncan’s multiple range test) when comparing values within the same ABA treatment.

It is known that the *pyr1 pyl1 pyl2 pyl4* quadruple mutant shows ABA insensitivity in ABA-induced ROS and NO production (Yin *et al*., 2013). It was observed that ABA-induced ROS and NO production was impaired in guard cells of *cch* and *rtl1* mutants, which was similar to that in the *pyr1 pyl1 pyl2 pyl4* quadruple mutant (Fig. 6A–D). The expression of some ROS metabolism-related genes was further tested. *RbohD* and *RbohF* encode two members of the NADPH oxidase family, which promote ROS production and are involved in ABA signalling likely downstream of OST1 (Kwak *et al.*, 2003; Acharya *et al.*, 2013). GPX1/2/5/6/7 encode five members of the glutathione peroxidase family, of which the expression is induced by environmental stresses (Sugimoto and Sakamoto, 1997; Rodriguez *et al.*, 2003); CAT1/2/3 encode three members of the catalase family (Chevalier *et al.*, 1992). The GPXs and CATs are responsible for ROS scavenging (Noctor *et al.*, 2002; Smykowski *et al.*, 2010). These enzymes are involved in maintaining ROS homeostasis in plant cells (Wang and Song, 2008). It was observed that ABA-induced expression of all these genes except for *RbohD* and *CAT3* significantly decreased in both the *pyr1 pyl1 pyl2 pyl4* and *cch* mutants (Fig. 6E–F), suggesting that both ROS production and scavenging processes are impaired in response to ABA in these mutants. These findings are consistent with the above-mentioned decline in the ABA sensitivity of ROS production of these mutants. Together, all the data suggest that CHLH/ABAR, like the PYR/PYL/RCAR receptors for ABA, acts upstream of ROS and NO in the ABA signalling pathway.

It was further tested, in the yeast one-hybrid system, whether the two important ABA-responsive transcription factors acting downstream of OST1, ABF4, and ABI5, may possibly bind the promoters of the ROS-metabolism-related genes to regulate their expression and ROS homeostasis. The results showed that neither ABF4 nor ABI5 binds to the promoter of *RbohD*, *RbohF*, *GPX1*, *GPX2*, *GPX5*, and *CAT2*, and seems to be unlikely to bind to the promoters of *CAT1* and *CAT3* (Supplementary Fig. S4). OST1 and ABAR did not associate with these promoters either, likely because they are not transcription factors (Supplementary Fig. S4). These data suggest that OST1 may not regulate ROS homeostasis downstream of ABAR and PYR/PYL/RCAR through ABA-responsive transcription factors such as ABF4 and ABI5, but is likely to regulate ROS-metabolism-related enzymes through direct phosphorylation at the post-translational level (Sirichandra *et al.*, 2009; Acharya *et al.*, 2013). It is not precluded, however, that OST1 phosphorylates transcription factors other than ABF4 and ABI5 to regulate ROS-metabolism-related gene expression, which needs further study.

### Phosphorylation of ABAR is independent of OST1 and ABA

**Fig. 7.**
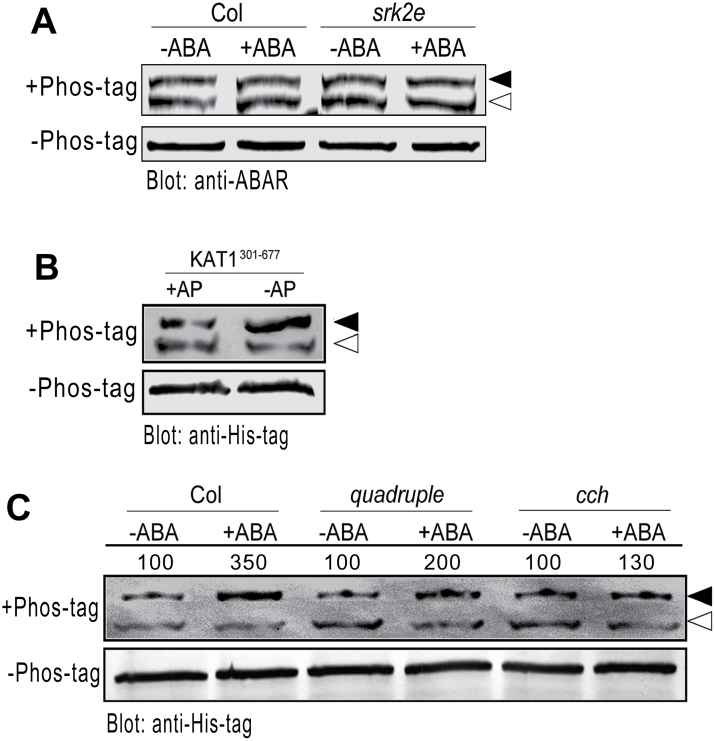
Phosphorylation assessment of the ABAR and KAT1 proteins by Phos-tag SDS-PAGE test. (A) ABAR phosphorylation in the *srk2e* mutant, detected with ABAR-immunoblot assay using Phos-tag SDS-PAGE test. Three-week-old plants of wild-type Col or *srk2e* mutant were treated with ABA-free (−ABA) or ABA-containing solution (+ABA) [50 μM (±)ABA] for 90min, then ABAR phosphorylation status was detected with anti-ABAR serum (+Phos-tag, top panel). Black and open arrows indicate the phosphorylated and dephosphorylated forms of ABAR, respectively. -Phos-tag SDS-PAGE (bottom panel) shows a loading control and indicates the ABAR protein with the expected molecular mass of ABAR (about 150kDa). The experiment was replicated five times with similar results. (B) Phosphorylation level of the His-tagged C-terminal domain of KAT1. The recombinant truncated KAT1 containing C-terminal region His301–Asn677 (KAT1^301–677^) was treated with alkaline phosphatase-containing (+AP) or AP-free buffer (−AP) for 6h. The phosphorylation of KAT1^301–677^ was assayed by a slower mobility in a Phos-tag SDS-PAGE that selectively retards phospho- KAT1^301–677^ (black arrow), where the His-tagged KAT1^301–677^ was detected by anti-His-Tag serum. Note that the AP treatment increases the amount of the dephosphorylation form of KAT1^301–677^ (open arrow). The bottom panel (indicated by -Phos-tag) shows a loading control. (C) ABA-activated phosphorylation of the KAT1^301–677^ protein in wild-type Col, *quadruple*, and *cch* mutant plants. Three-week-old plants were treated with ABA-free (−ABA) or 50 μM (±)ABA-containing medium (+ABA) for 90min. The phosphorylation was assayed in the extracted total protein, using KAT1^301–677^ as a substrate, and the His-tagged KAT1^301–677^ was detected by anti-His-Tag serum (top panel). Black and open arrows indicate phosphorylated and dephosphorylated KAT1^301–677^, respectively. Note that the amounts of phosphorylated KAT1^301–677^ protein are apparently comparable among the three genotypes Col, *quadruple*, and *cch* with ABA-free treatment (−ABA). The amounts of the phosphorylated KAT1^301–677^ protein with ABA treatment were evaluated by scanning the protein bands, and relative band intensities, normalized relative to the intensity of the ABA-free treatment sample (as 100%), are indicated by numbers above the bands. Note that, in comparison with Col, the levels of the ABA-activated phosphorylated KAT1^301–677^ protein in *quadruple* and *cch* decrease. -Phos-tag SDS-PAGE (bottom) shows a loading control and indicates the expected molecular mass of His-tagged KAT1^301–677^ (about 60kDa). The experiment was repeated five times with similar results.

Upon activation by ABA, OST1 modulates the activities of downstream effectors to regulate stomatal movement by phosphorylation (Sato *et al.*, 2009; Sirichandra *et al.*, 2009; Geiger *et al.*, 2009, 2010; Lee *et al.*, 2009, 2013; Brandt *et al.*, 2012; Acharya *et al.*, 2013; Imes *et al.*, 2013; Osakabe *et al.*, 2013; Liang and Zhang, 2014). A recent report suggests that ABAR may be phosphorylated (Wang *et al.*, 2013*a*). It was tested whether ABAR is a substrate of OST1. In the Phos-tag SDS-PAGE assay, in which the phosphorylated proteins with the phosphate group bound to the divalent metal ions decreases the migration speed, separated ABAR bands were observed on the gels (Fig.7A), indicating that ABAR was phosphorylated by the protein kinase (Fig. 7A), which is consistent with a previous observation (Wang *et al.*, 2013*a*). However, the amount of phosphorylated ABAR in wild-type Col plants was comparable with that in the *srk2e* mutant, and ABA treatment did not change the amount of phosphorylated ABAR in wild-type Col plants or in the *srk2e* mutant (Fig. 7A), suggesting that phosphorylation of ABAR is independent of OST1 and ABA.

### ABA-induced activation of K^+^ channel KAT1 phosphorylation is impaired in both *cch* and *pyr1 pyl1 pyl2 pyl4* mutants

The inward K^+^ channel KAT1, of which the activity is inhibited by ABA, is a direct phosphorylation target of OST1 (Sato *et al.*, 2009; Acharya *et al.*, 2013). The inward-rectifying K^+^ and anion channel responses to ABA were impaired in the *pyr1 pyl1 pyl2 pyl4* quadruple mutant (Wang *et al.*, 2013*b*), consistent with the idea that KAT1 is regulated by OST1 that acts downstream of PYR/PYL/RCAR receptors. However, there is no evidence that KAT1 phosphorylation is affected in the *pyr1 pyl1 pyl2 pyl4* quadruple mutant. Recombinant truncated KAT1 protein containing the C-terminal region (His301–Asn677, KAT1^301–677^; Supplementary Fig. S5) was used as a substrate to assess whether ABAR is involved in the regulation of KAT1 phosphorylation. This C-terminal region of KAT1 was identified as the phosphorylation domain that may be phosphorylated by OST1 independently of other domains (Sato *et al.*, 2009). It was found that the KAT1^301–677^ truncated protein produced in *E. coli* was phosphorylated by protein kinases in *E. coli.* (upper band, Fig.7B), and the phosphatase treatment increased the dephosphorylation form of KAT1^301–677^ (lower band, Fig. 7B); therefore, the phosphatase-treated KAT1^301–677^ was used in the KAT1^301–677^ phosphorylation assays in total proteins prepared from different genotypes. The KAT1^301–677^ phosphorylation activity was shown to be enhanced by ABA (Fig. 7C), which is consistent with the idea that KAT1 is phosphorylated by the ABA-activated OST1 kinase (Mustilli *et al.*, 2002; Yoshida *et al.*, 2002, 2006; Belin *et al.*, 2006; Fujii and Zhu, 2009; Sato *et al.*, 2009; Acharya *et al.*, 2013). This ABA-induced activation of KAT1^301–677^ phosphorylation was observed in all the genotypes including wild-type Col, *cch*, and *pyr1 pyl1 pyl2 pyl4* quadruple mutants, of which the levels, however, significantly decreased in both the *pyr1 pyl1 pyl2 pyl4* and *cch* mutants (Fig. 7C).

## Discussion

### OST1 interacts with, and functions downstream of, ABAR in guard cell signalling in response to ABA

A combination of yeast two-hybrid system, pull down, LCI, CoIP, and SPR assays showed consistently that ABAR interacts directly with OST1 (Fig. 1), a critical signalling component in the PYR/PYL/RCAR-mediated ABA signalling pathway in guard cells (Mustilli *et al.*, 2002; Yoshida *et al.*, 2002; 2006; Sato *et al.*, 2009; Sirichandra *et al.*, 2009; Brandt *et al.*, 2012; Acharya *et al.*, 2013; Imes *et al.*, 2013; Osakabe *et al.*, 2013). Although ABAR/CHLH is a chloroplast protein, it spans the chloroplast envelope with its N and C termini exposed to the cytosol (Shang *et al.*, 2010). The C-terminus of ABAR binds to a group of WRKY-domain transcription repressors to regulate expression of ABA-responsive genes (Shang *et al.*, 2010; Liu *et al.* 2013; Yan *et al.*, 2013). OST1, localized to cytosolic and nuclear spaces (Nakashima *et al.*, 2009; Sirichandra *et al.*, 2010; Ding *et al.*, 2015), interacts with the C-terminal half, but not N-terminal half or middle section of ABAR (Fig. 1). This suggests that the interaction between ABAR and OST1 is likely to take place in the cytosol, which is similar to that between ABAR and the WRKY transcription factors (Shang *et al.*, 2010). However, the cytosolic localization of the interaction between ABAR and OST1 should be confirmed in the future using other techniques, such as bimolecular fluorescence complementation system in *Arabidopsis* protoplasts.

Neither mutation nor over-expression of the ABAR gene affects significantly ABA-insensitive phenotypes of stomatal movement in the OST1 knockout mutant allele *srk2e*. However, over-expression of the *OST1* gene suppresses ABA-insensitive phenotypes of the ABAR mutant allele *cch* in stomatal movement (Figs 2–4). These genetic data demonstrate that OST1 functionally interacts with, and acts downstream of, ABAR in ABA signalling in guard cells. In addition, ABAR protein is shown to be phosphorylated, but independently of the OST1 protein kinase, which is consistent with the idea that ABAR functions upstream of OST1 (Fig. 7). These genetic and biochemical findings allow a functional link between ABAR and OST1 to be established in guard cell signalling in response to ABA.

### How does ABAR functionally interact with OST1 in ABA signalling in guard cells?

Owing to technical difficulties, the phosphorylation or kinase activity of OST1 when the function of ABAR is lesioned in *cch* or *rtl1* mutants was not determined; however, is important to understand the functional interaction between the two proteins and this needs to be tested with improved techniques in the future. However, this study has provided several lines of evidence supporting that ABAR, functioning upstream of OST1, shares, at least partly, downstream signalling components with the PYR/PYL/RCAR receptors in guard cells. Whereas the responses of ABA to induce stomatal closure and to inhibit stomatal opening are impaired in two ABAR mutant alleles, *cch* and *rtl1*, only the ABA response to induce stomatal closure was impaired in the *pyr1 pyl1 pyl2 pyl4* quadruple mutant that shows weak to wild-type response in ABA-inhibited stomatal opening (Fig. 5). This phenomenon in the *pyr1 pyl1 pyl2 pyl4* quadruple mutant was previously observed and explained by functional redundancy of the PYR/PYLRCAR family proteins (consisting of 14 members) or existence of other receptors involved in this ABA response in ABA-inhibited stomatal opening (Yin *et al*., 2013). Nevertheless, similar to the *pyr1 pyl1 pyl2 pyl4* quadruple mutant, the *cch* and *rtl1* mutants show lesioned ABA responses in ABA-induced ROS and NO production, as well as in ABA-activated phosphorylation of a K^+^ inward channel KAT1 in guard cells (Figs 6 and 7), supporting the idea that ABAR shares these downstream signalling regulators with PYR/PYL/RCAR receptors in guard cell signalling. Therefore, ABAR functions to directly interact with OST1 to regulate downstream signalling components such as ROS, NO, and KAT1 in a mechanism similar to the PYR/PYL/RCAR-mediated ABA signalling pathway in guard cells where PYR/PYL/RCAR receptors regulate OST1 through clade A PP2Cs to interact with ROS and NO messengers to modulate the function of the inward K^+^ channels such as KAT1 (Pei *et al.*, 2000; Zhang *et al.*, 2001; Mustilli *et al.*, 2002; Neill *et al.*, 2002; Garcia-Mata *et al.*, 2003; Kwak *et al.*, 2003; Bright *et al.*, 2006; Acharya *et al.*, 2013; Wang *et al.*, 2015).

In addition, it was previously reported that ABA inhibits BL-mediated stomatal opening in part via ABA-activated guard cell H^+^-ATPase phosphorylation mediated by OST1 (Hayashi and Kinoshita, 2011; Hayashi *et al.*, 2011), and ABAR/CHLH regulates guard cell H^+^-ATPase phosphorylation, which may be a mechanism to explain the role of ABAR in regulating ABA-induced inhibition of BL-induced stomatal opening (Tsuzuki *et al.*, 2013). In this regard, ABAR is likely to modulate H^+^-ATPase phosphorylation through OST1 in guard cells, which may be a key process to regulate inward ion flux across the plasma membrane of guard cells to affect stomatal opening. Further investigations will be needed to elucidate cooperation or crosstalk of ABAR-mediated signalling with PYR/PYL/RCAR-mediated signalling, in which the genetic interactions between ABAR and PYR/PYL/RCAR in guard cell signalling in response to ABA, for example, need to be determined in the future. These studies will help to understand the complex ABA signalling network.

## Supplementary data

Supplementary data are available at *JXB* online.

Fig. S1. Interaction of ABAR with OST1 tested with the SPR system.

Fig. S2. Identification of the GFP-tagged ABAR^631–1381^ expression in the *srk2e* mutant plants.

Fig. S3. Phenotypes of the Myc-tagged OST1-over-expression lines.

Fig. S4. Yeast one-hybrid assays to test possible interactions of ABF4, ABI5, OST1, or ABAR with the promoters of ROS-metabolism-related genes *RbohD*, *RbohF*, *GPX1*, *GPX2*, *GPX5*, *CAT1*, *CAT2*, and *CAT3*.

Fig. S5. Purified recombinant truncated KAT1 protein (KAT1^301–677^, aa 301–677) tested with an SDS-PAGE gel.

Table S1. PCR primers used in this study.

Supplementary Data
