# Supplementary material for: A link between magnesium-chelatase H subunit and sucrose nonfermenting 1 (SNF1)-related protein kinase SnRK2.6/OST1 in Arabidopsis guard cell signalling in response to abscisic acid
Source: J Exp Bot. 2015 Jul 13;66(20):6355–69. doi: 10.1093/jxb/erv341 (PMC4588886; doi:10.1093/jxb/erv341)
Supplement: Supplementary Data [file supp_66_20_6355__index.html]

A link between magnesium-chelatase H subunit and sucrose nonfermenting 1 (SNF1)-related protein kinase SnRK2.6/OST1 in Arabidopsis guard cell signalling in response to abscisic acid — Supplementary Data 

# A link between magnesium-chelatase H subunit and sucrose nonfermenting 1 (SNF1)-related protein kinase SnRK2.6/OST1 in *Arabidopsis* guard cell signalling in response to abscisic acid

## Supplementary Data

Data files

- Supplementary Data - Supplementary Data
