# Supplementary material for: A link between magnesium-chelatase H subunit and sucrose nonfermenting 1 (SNF1)-related protein kinase SnRK2.6/OST1 in Arabidopsis guard cell signalling in response to abscisic acid
Source: J Exp Bot. 2015 Jul 13;66(20):6355–69. doi: 10.1093/jxb/erv341 (PMC4588886; doi:10.1093/jxb/erv341)
Supplement: Supplementary Data [file supp_erv341_Supplementary_Data_3ndRevision.pdf]

Supplementary Data

Supplementary materials and methods

Surface plasmon resonance (SPR) assay

Surface plasmon resonance (SPR) assay was performed using a Biacore T200 equipment (GE Healthcare, Piscataway, NJ, USA). Recombinant truncated ABAR/CHLH protein was dissolved in 10 mM sodium acetate (pH 5) with final concentration of 50 μg/mL. A certified CM5 sensor chip with carboxyl groups on its surface was used to bind ABAR protein via -NH_2 bond. The surface of flow cell 2 was activated for 7 min with a 1:1 mixture of 0.1 M NHydroxysuccinimide (NHS) and 0.1 M 1-ethyl-3-(3- dimethylaminopropyl) carbodiimide hydrochloride (EDC) at a flow rate of 10 μL/min; flow cell 1 was left blank to serve as a reference surface. The surface was then blocked with a 7 min injection of 1 M ethanolamine, pH 8.0. To collect kinetic and affinity binding data, the analyte OST1 protein in the running buffer (10 mM HEPES, 150 mM NaCl, 30 mM EDTA, and 0.005 % v/v surfactant P20, pH 7.4) was injected over flow cell 1 and flow cell 2 at different concentrations (500 nM, 50 nM, 125 nM, 62.5 nM, 31.25 nM) using glycine solution (pH 2.5) for regeneration. Data were collected and globally fitted to steady-state model available within Biacore Evaluation software v1.01.

Yeast one-hybrid assays

Yeast one-hybrid assays were performed as previously described (Shang et al., 2010). The yeast strain AH109 was chosen to be host strain. The primers used for constructing the related plasmids were listed in supplemental table 1. Open reading frame of *OST1*, *ABF4*, *ABI5* and cDNA sequence encoding the C-terminal half of ABAR (amino acid residues from 692 to 1381, $ABAR_{c690}$) were constructed into pGADT7 prey vector, whereas the promoter fragment of *RbohD*, *RbohF*, *GPX1*, *GPX2*, *GPX5*, *CAT1*, *CAT2* and *CAT3* were cloned into the bait vector pHIS2. Protein linked pGADT7 prey vector and promoter linked pHIS2 bait vector were co-transformed into yeast cells. Yeast cells co-transformed with the empty pGADT7 vector and pHIS2 harbouring the corresponding promoter were used as negative controls. Yeast cells co-transformed with P53-pGADT7 and pHIS2 harbouring the promoter of P53 was used as positive control. Transformed yeast cells were first grown in SD-2 medium (lacking tryptophan and leucine) for 3 days to ensure that the yeast cells were successfully co-transformed and positive clones were cultured in SD-2 liquid medium overnight to OD600 of 0.1 and diluted in a 10× dilution series. 8μL yeast cells was dropped on SD-2 and SD-3 medium (lacking Trp, Leu, His) supplemented with different concentrations (20 mM, 40 mM, 60 mM, 80 mM, 100 mM, 120 mM) of 3-aminotriazole (3-AT; Sigma). The plates were then incubated for 3 d at 30 °C prior to photograph.

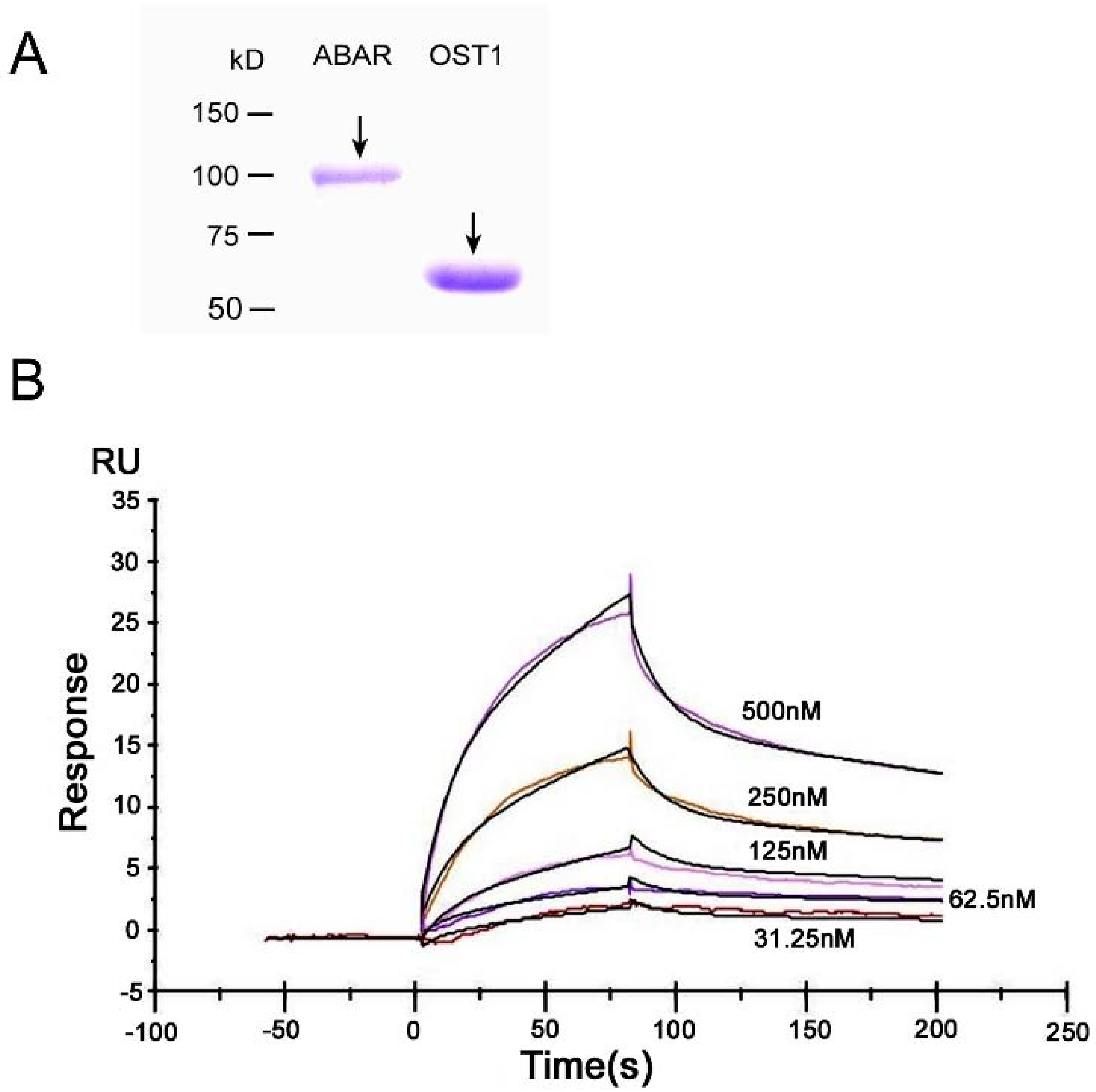

Fig. S1. Interaction of ABAR with OST1 tested with surface plasmon resonance (SPR) system. **(A)** Purified recombinant OST1 and C-terminal half of ABAR (amino acid residues 681-1381), tested by SDS-PAGE gel. kD indicates molecular mass. **(B)** SPR assay: C-terminal half of ABAR binds OST1 directly. The recombinant truncated ABAR protein was bound to sensor chip, and then different concentrations of OST1 (500 nM, 250 nM, 125 nM， 62.5 nM, and 31.25 nM) were used to detect the interaction. The corresponding curves show that the interaction of OST1 with ABAR is dose-dependent, where the disassociation constant (Kd) approximately equal to 7.145×10^{-8} M. RU, response unit. The experiments were repeated independently five times with the similar results. The experimental procedures are presented in Supplementary materials and methods section.

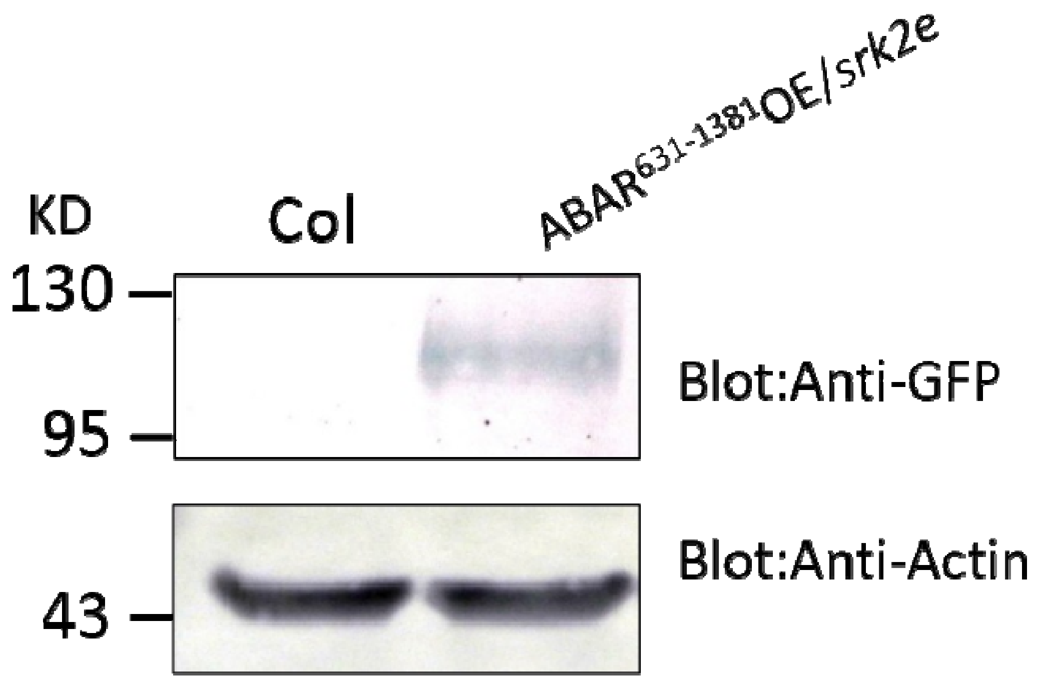

Fig. S2. Identification of the GFP-tagged ABAR$^{631\text{-}1381}$ expression in the *srk2e* mutant plants. The GFP-tagged ABAR$^{631\text{-}1381}$ expression (ABAR$^{631\text{-}1381}$OE/*srk2e*) was detected by immunoblotting with anti-GFP serum (Blot: Anti-GFP). The total protein extracted from the wild-type Col plants was used as a negative control. Actin was used as a loading control (Blot: Anti-Actin). kD indicates molecular mass.

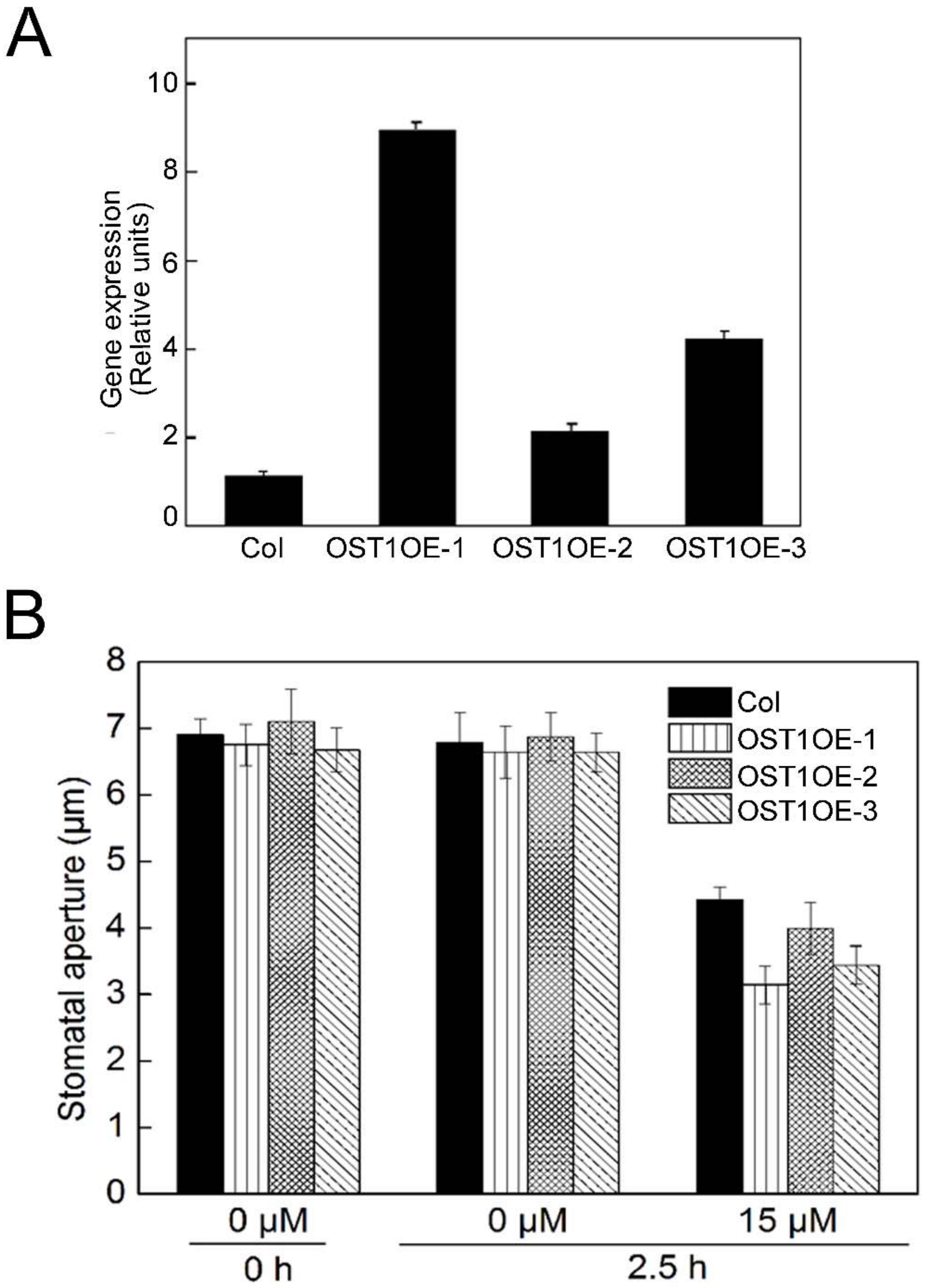

Fig. S3. Phenotypes of the Myc-tagged *OST1*-overexpression lines. **(A)** Test of the expression levels of the *OST1* gene in the three transgenic lines by real-time PCR analysis. **(B)** The *OST1*-overexpression lines displayed ABA-hypersensitive phenotype in ABA-induction of stomatal closure. For quantitative real-time PCR analysis, see Supplementary materials and methods section.

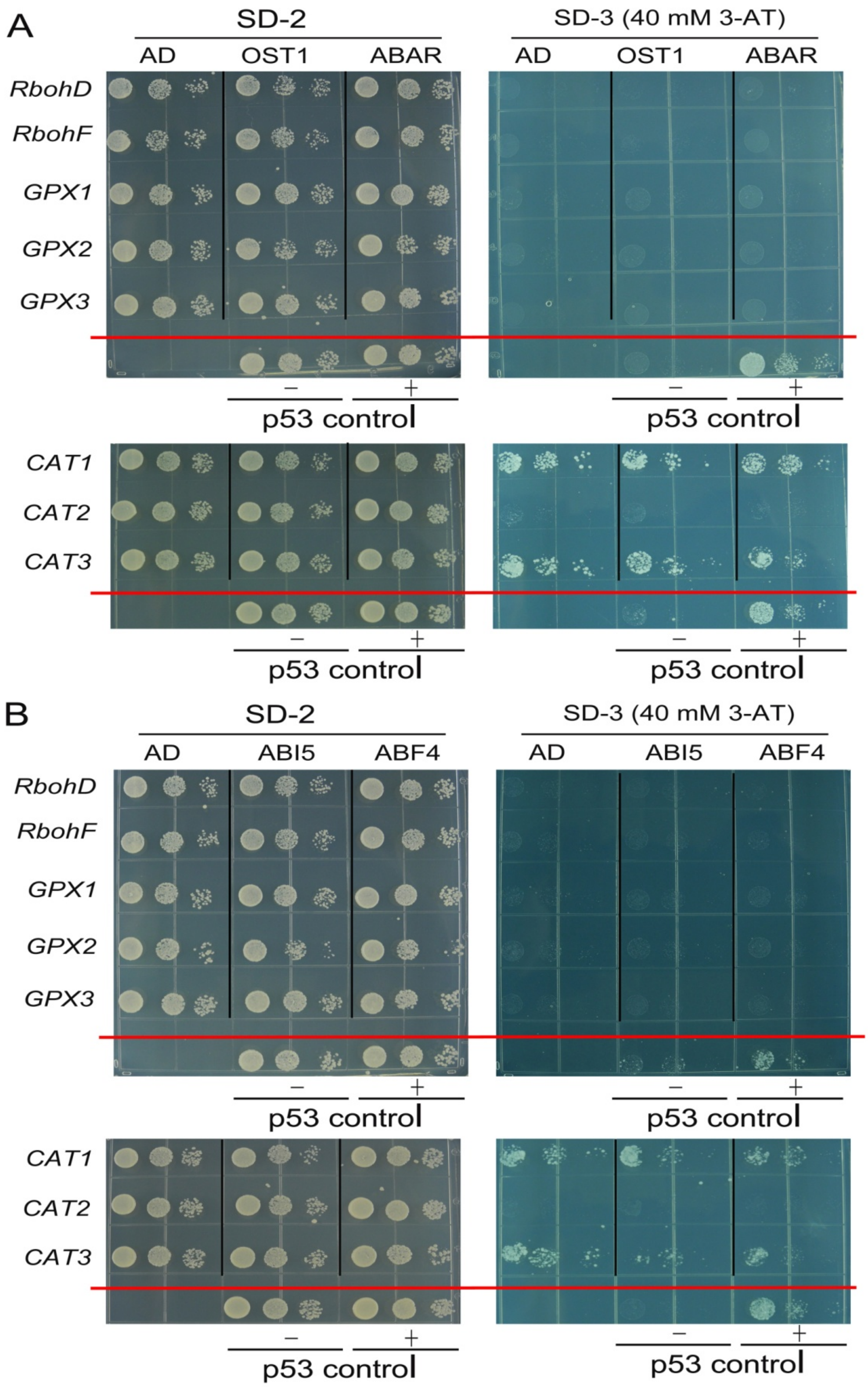

Fig. S4. Yeast one-hybrid assays to test possible interactions of ABF4, ABI5, OST1 or ABAR with the promoters of ROS-metabolism-related genes *RbohD*, *RbohF*, *GPX1*, *GPX2*, *GPX5*, *CAT1*, *CAT2* and *CAT3*. The data showed

that OST1, $ABAR_{c690}$, ABF4 or ABI5 do not bind to the promoter of *RbohD*, *RbohF*, *GPX1*, *GPX2*, *GPX5*, and *CAT2*, and are unlikely to bind to the promoter of *CAT1* and *CAT3*. **(A)** Yeast cells co-transformed with pGADT7 prey vector harboring open reading frame (ORF) of *OST1* or *ABAR$_{c690}$* coding for the C-terminal of ABAR, and pHIS2 bait vector harboring promoter sequence of *RbohD*, *RbohF*, *GPX1*, *GPX2*, *GPX5* or *CAT2* grew well in SD-2 medium and failed to grow in SD-3 medium with 40 mM 3-AT. These data suggest that neither OST1 nor $ABAR_{c690}$ binds to the promoter of these genes. **(B)** Yeast cells co-transformed with pGADT7 prey vector harboring ORF of *ABI5*, *ABF4*, and pHIS2 bait vector harboring promoter sequence of *RbohD*, *RbohF*, *GPX1*, *GPX2*, *GPX5*, and *CAT2* grew well in SD-2 medium, and failed to grow in SD-3 medium, suggesting that neither ABI5 nor ABF4 binds to the promoter of these genes.

Yeast cells co-transformed by the combinations of empty pGADT7 vector, *OST1*, *ABAR$_{c690}$ ABI5* or *ABF4*, with promoters of *CAT1,CAT3*, respectively, grew in SD-3 medium even in the presence of 120 mM 3-AT, due to the self-activation phenomenon in this yeast one hybrid system with these combinations (**A, B)**. Therefore, it cannot be determined with the yeast one hybrid system whether OST1, $ABAR_{c690}$, ABI5 or ABF4 interact with the promoters of *CAT1* or *CAT3*.

Transformation with empty vectors pGADT7 and pHIS2 harboring the corresponding promoters were used as negative controls. Given that P53 protein binds to its own promoter, co-transformation of pHIS2 harboring the promoter of *P53* with pGADT7 harboring *P53* (below the red lines, p53 control , +) were used as positive control. Transformation of pHIS2 harboring the promoter of *P53* with pGADT7 harboring *P53* or empty pGADT7 were used as negative control (below the red lines, p53 control , –). All the experiments were repeated three times with the same results.

Abbreviations: AD, empty vector pGADT7; SD-2, medium lacking Trp and Leu; SD-3 medium lacking Trp, Leu and His.

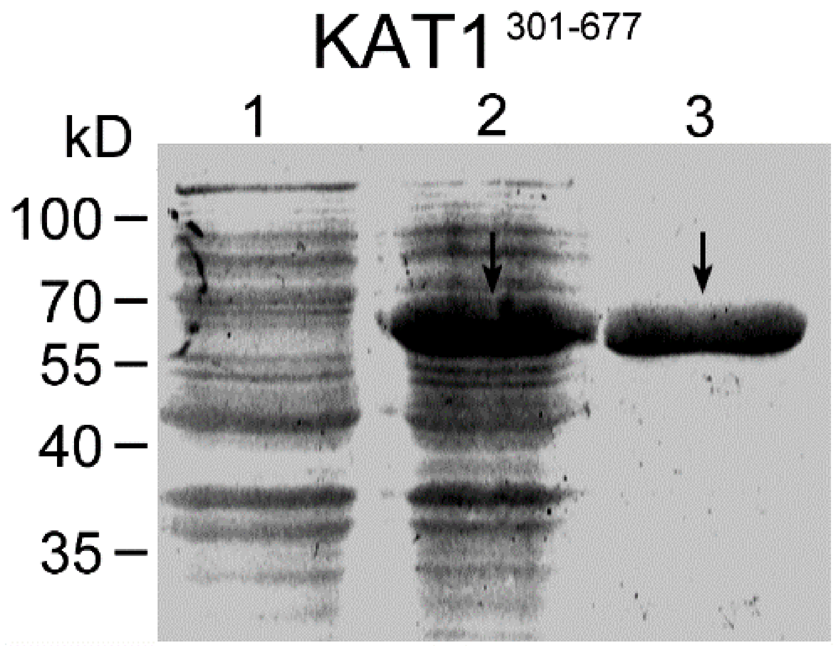

Fig. S5. Purified recombinant truncated KAT1 protein (KAT1$^{301\text{-}677}$, amino acid residues 301-677) tested in SDS-PAGE gel. Lane 1, total protein of the un-induced control cells. Lane 2, total protein of the induced cells. Lane 3, purified protein. kD indicates molecular mass.

Table S1. PCR primers used in this study.

1. **Primers for yeast two hybrid assay.** BD, the binding domain in the bait vector pGBKT7; AD, the activation domain in the prey vector pGADT7.

Primer Name	Sequence（5’-3’）
**ABAR$_{1\text{-}691}$-BD-Forward**	CCGGAATTCATGGCTTCGCTTGTGTATTCTCC
**ABAR$_{1\text{-}691}$-BD-Reverse**	ACGCGTCGACGATAAGACTGTCGGGAAAAC
**ABAR$_{692\text{-}941}$-BD-Forward**	GGAATTCGGGAACATTCCCAATG
**ABAR$_{692\text{-}941}$-BD-Reverse**	ACGCGTCGACGAAACCAAACACTGTTCTGAGCT
**ABAR$_{692\text{-}1381}$-BD-Forward**	GGAATTCGGGAACATTCCCAATG
**ABAR$_{692\text{-}1381}$-BD-Reverse**	ACGCGTCGACTCGATCGATCCCTTCGATCTTG
**OST1-AD-Forward**	CGGAATTCATGGATCGACCAGCAGTGAG
**OST1-AD-Reverse**	CGGGATCCTCACATTGCGTACACAATC

2. **Primers for yeast one hybrid assay**

Primer Name	Sequence（5’-3’）
**RBOHD-pHIS2-Forward**	TCCCCCGGG CAAGAAACCAAAGTAGAAGGTCAGTGC
**RBOHD-pHIS2-Reverse**	CGACGCGTCGAATTCGAGAAACCAAAAAGAT
**RBOHF-pHIS2-Forward**	TCCCCCGGGGGTATTACTTGAACTAACGTCATCCGAG
**RBOHF-pHIS2-Reverse**	CGACGCGTAGATCCAAAGTCGGAATTCAAAGAG
**GPX1- pHIS2-Forward**	TCCCCCGGGTTGTGAAAGGAGAAAAGAG
**GPX1- pHIS2-Reverse**	CGACGCGTCGTACGAAGAAGATGAAGTA
**GPX2- pHIS2-Forward**	TCCCCCGGGGCACCGACCAACTGCTAAC
**GPX2- pHIS2-Reverse**	CGACGCGTGGTTGATAAATGTTATAAGATCAGAAT
**GPX5- pHIS2-Forward**	TCCCCCGGGAAGGTCTATCATACCCTCACA
**GPX5- pHIS2-Reverse**	CGACGCGTTAACAAACAAACCCATCC
**CAT1-pHIS2-Forward**	TCCCCCGGGACCCCTCAAAGTTTGATC
**CAT1-pHIS2-Reverse**	CGACGCGTGATGCTTGAAGACAATTTTGATC
**CAT2-pHIS2-Forward**	TCCCCCGGGAAATAATCTTATATTGAAGAAG
**CAT2-pHIS2-Reverse**	CGACGCGTTCTGAGAAAGCAGCATCTCTT
**CAT3-pHIS2-Forward**	TCCCCCGGGAATCAAAAATTGTGGATCAGTT
**CAT3-pHIS2-Reverse**	CGACGCGTGGTGATGATAGAAGGTTGATGA

**ABAR-pGADT7-Forward**	GGAATTC GGGAACATTCCCAATGTCTACTATT
**ABAR-pGADT7-Reverse**	CGGGATCCCTCGATCGATCCCTTCGATCTTG
**ABF4-pGADT7-Forward**	GGAATTCATGGGAACTCACATCAATTTC
**ABF4-pGADT7-Reverse**	CGGGATCCCCCATGGTCCGGTTAATGTCC
**ABI5-pGADT7-Forward**	GGAATTCATGGTAACTAGAGAAACGAAG
**ABI5-pGADT7-Reverse**	CGGGATCCCGAGTGGACAACTCGGGTTC

3. **Primers for identification of the mutants**

Primer Name	Sequence（5'-3'）
**LBb1.3**	ATTTTGCCGATTTCGGAAC
***srk2e*-LP**	CTCGAGCTTTAAGTCTCGGTG
***srk2e*-RP**	TCGCAAAGAGACAGAGGAAAG
***cch*-Forward**	AGGCTGCTTTTCTCCAAGTCAGCAAGGC
***cch*-Reverse**	TTGGCATAACTTCTCCTCTTTG

4. **Primers for production of recombinant protein**

Primer Name	Sequence（5'-3'）
**OST1(pET48b)-Forward**	CGGGATCCGATGGATCGACCAGCAGTG
**OST1(pET48b)-Reverse**	GCGTCGACTCACATTGCGTACACAATC
**$ABAR_{681-1381}$-Forward**	GGAATTCATGAGTGATGCTTGTTTTC
**$ABAR_{681-1381}$-Reverse**	ACGCGTCGACTTATCGATCGATCCCTTCGATCTTG
**$KAT1^{301-677}$-Forward**	CGGGATCCGCATTGGACTAGCCGAACCAG
**$KAT1^{301-677}$-Reverse**	GCGTCGACATTTGATGAAAAATACAAATG

5. **Primers for firefly luciferase complementation imaging (LCI)**. Nluc and Cluc, N-terminal and C-terminal half of the luciferase (Luc), respectively.

Primer Name	Sequence（5'-3'）
**ABAR-NLuc-Forward**	GGGGTACCATGGCTTCGCTTGTGT
**ABAR-NLuc-Reverse**	ACGCGTCGACTCGATCGATCCCTTC
**OST1-CLuc-Forward**	GGGGTACCATGGATCGACCAGCAGTGAG
**OST1-CLuc-Reverse**	CGGGATCCTCACATTGCGTACACAATC

6. **Primers for quantitative real-time PCR (qRT-PCR)**

Primer Name	Sequence（5’-3’）
**ACTIN-qRT-Forward**	GGTAACATTGTGCTCAGTGGTGG
**ACTIN-qRT-Reverse**	AACGACCTTAATCTTCATGCTGC
**OST1-qRT-Forward**	TGGAGTTGCGAGATTGATGAGAG
**OST1-qRT-Reverse**	CCTGTGGTTGATTATCTCCCTTTTT
**RbohD -qRT-Forward**	CTAGCTTTGGATTTTTCTCGA
**RbohD -qRT-Reverse**	GTAACCAACAAAACGGTAGGG
**RbohF -qRT-Forward**	CTAAAAGACAAGAAGGAAGAAGCC
**RbohF -qRT-Reverse**	CTTCTAGAGTCTCCATCTCATTTGC
**CAT1-qRT-Forward**	TCATCGGGAAGGAGAACA
**CAT1-qRT-Reverse**	GAAACCAAACCGTAAGAG GAGCA
**CAT2-qRT-Forward**	TCAAACCCGTGTCTTCTCCTATGCCG
**CAT2-qRT-Reverse**	AACAGACAGCAGGCGGAGTTGGATAC
**CAT3-qRT-Forward**	TGCCAAATCTGGAAAAGTTC
**CAT3-qRT-Reverse**	GGTCACATCAAGTGGGTCAAAGTCAAAC
**GPX1-qRT-Forward**	GTCTCCGGTAACCAAAAATG
**GPX1-qRT-Reverse**	GACGAGAAAGGTTGCTGAGG
**GPX2-qRT-Forward**	AAACTGCGTTGGGACAGG-
**GPX2-qRT-Reverse**	CCCATGAAAAGACATCGAATAC
**GPX5-qRT-Forward**	CAAAACGCTGCACCAGTC
**GPX5-qRT-Reverse**	GACCATCTTTGCCGACCAAG
**GPX6-qRT-Forward**	GATGTTAACGGTGACAAAGCTG
**GPX6-qRT-Reverse**	TTGGTGCGAAACGATCG
**GPX7-qRT-Forward**	TCGGCCCATCATTGAGATTC
**GPX7-qRT-Reverse**	CTGCAGCCCTTGCATAGAC

7. Primers for generating the *OST1* over-expression lines

Primer Name	Sequence（5’-3’）
**OST1OE-Forward**	GCTCTAGAATGGATCGACCAGCAGTGAG
**OST1OE-Reverse**	GCGTCGACGCATTGCGTACACAATC
